# *Aspergillus niger* as a Secondary Metabolite Factory

**DOI:** 10.3389/fchem.2021.701022

**Published:** 2021-07-30

**Authors:** Ronglu Yu, Jia Liu, Yi Wang, Hong Wang, Huawei Zhang

**Affiliations:** ^1^ School of Pharmaceutical Sciences, Zhejiang University of Technology, Hangzhou, China; ^2^ Key Laboratory of Marine Fishery Resources Exploitment and Utilization of Zhejiang Province, Hangzhou, China

**Keywords:** *Aspergillus niger*, secondary metabolite, bioactivity, biosynthesis, application

## Abstract

*Aspergillus niger*, one of the most common and important fungal species, is ubiquitous in various environments. *A. niger* isolates possess a large number of cryptic biosynthetic gene clusters (BGCs) and produce various biomolecules as secondary metabolites with a broad spectrum of application fields covering agriculture, food, and pharmaceutical industry. By extensive literature search, this review with a comprehensive summary on biological and chemical aspects of *A. niger* strains including their sources, BGCs, and secondary metabolites as well as biological properties and biosynthetic pathways is presented. Future perspectives on the discovery of more *A. niger*-derived functional biomolecules are also provided in this review.

## Introduction

*Aspergillus*, one sizeable genus belonging to Aspergillaceae family, comprises as many as 492 species registered on the database of the National Center for Biotechnology Information (NCBI) to date. Its section *Nigri* is an important group of species, and the *A. niger* aggregate represents its most complicated taxonomic subgroup with eight morphologically indistinguishable taxa (Perrone et al., 2011). Owing to superior adaptability and survivability, *A. niger* is ubiquitous in nature, including in terrestrial soil (Xie et al., 2006), ocean (Li et al., 2016; Uchoa et al., 2017), the Arctic (Singh et al., 2011), and space. It also occupies a wide spectrum of habitats in plants and animals such as herb (Shreelalitha and Sridhar, 2015; Manganyi et al., 2018), shrub (Kaur et al., 2015; Liu et al., 2016), tree (Soltani and Moghaddam, 2014; Wang et al., 2019), lichen (Elissawy et al., 2019), shrimp (Liu et al., 2013; Fang et al., 2016), and marine sponge (Takano et al., 2001; Hiort et al., 2004). *A. niger* strain grows well in various media with different carbon sources, including glucose, bran, maltose, xylan, xylose, sorbitol, and lactose (Toghueo et al., 2018). However, its metabolism is remarkably affected by culture conditions, such as medium composition and fermentation mode.

**FIGURE 1:**
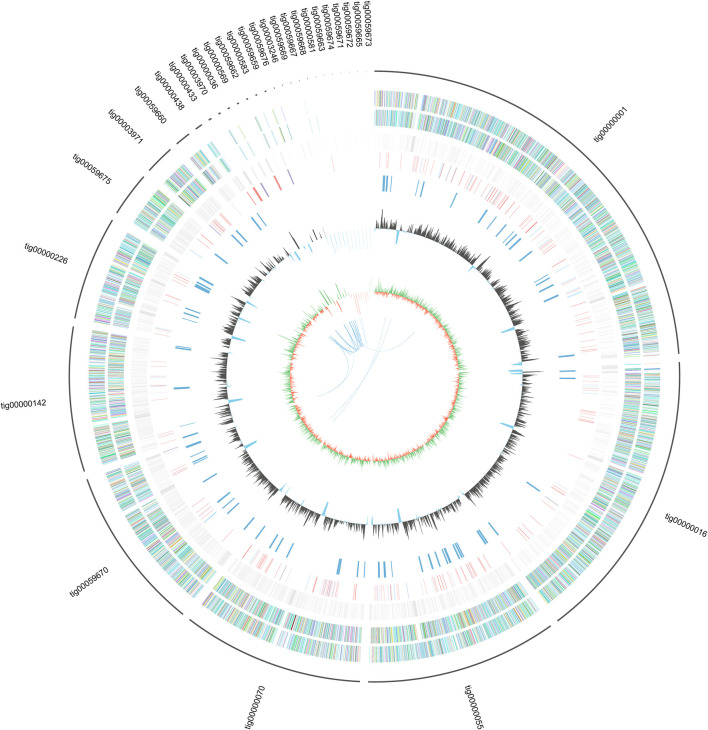
General genome features of marine strain *Aspergillus niger* L14 (From the inside out: In-paralog pairs; GC skew, the green part represent positive value while the orange part represent negative value; G + C%; SMs gene clusters; ncRNA; repeat; minus strand coding sequence (CDS) annotation; plus strand CDS annotation; scaffolds).

**FIGURE 2:**
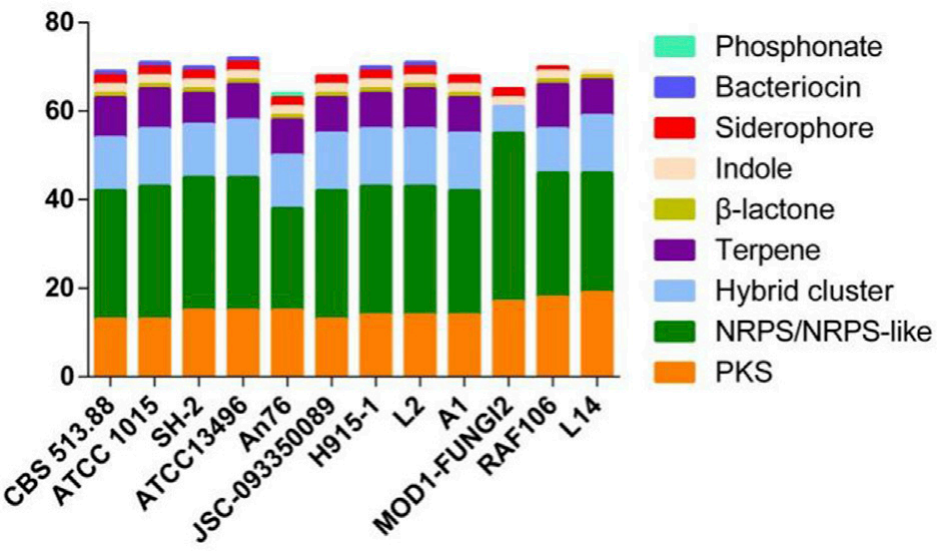
Biosynthetic gene clusters of secondary metabolites of 12 *A. niger* strains.

The genome features of strain L14 are summarized in a polycyclic graph (Figure 1), which consists of in-paralog pair, GC skew, widely, SM biosynthetic gene cluster (BGC), ncRNA, repeat, strand coding sequence (CDS) annotation, and scaffold. There are some in-paralog pairs between different scaffolds, and SM BGCs and CDS distributed widely in genome. As shown in Table 1, genome sizes of WT *A. niger* strains range from 33.8 to 36.1 Mb. Their G + C% and gene numbers are closely similar, while the numbers of scaffolds are different owing to various sequencing and assembling manners. The antibiotics and Secondary Metabolite Analysis Shell (antiSMASH) results indicated that each WT *A. niger* strain harbors at least 20 cryptic SM BGCs, including PKS, NRPS, NRPS-like, and their hybrids (Figure 2 and Supplementary Table S1) (Blin et al., 2019). These BGCs involving in indole and terpene biosynthesis are ubiquitous and have great potential to synthesize therapeutical agents and pesticides, such as AbT1, azanigerone A, fusarin, ferrichrome, nidulanin A, melanin, TAN-1612, yanuthone D, and aflavarin (Supplementary Table S2).

**TABLE 1:** General genomic features of 12 *Aspergillus niger* strains from NCBI database.

Strain	Genome size (Mb)	G + C%	Scaffold	Gene	tRNA	Protein-coding genes	Isolation source	Assembly ID
ATCC 1015	34.8	50.3	24	10947	–	10950	–	GCA_000230395.2
CBS 513.88	34.0	50.4	20	10828	263	14165	–	GCA_000002855.2
SH-2	34.6	50.3	349	–	–	–	Soil	GCA_000633045.1
ATCC 13496	35.7	49.5	133	12468	273	12194	–	GCA_003344705.1
An76	34.9	49.4	669	10373	–	10373	Soil	GCA_001515345.1
JSC-093350089	36.1	49.5	223	–	–	–	International space station environmental surface	GCA_001931795.1
H915-1	36.0	49.2	30	–	–	–	Soil	GCA_001741905.1
L2	36.4	49.2	30	–	–	–	Soil	GCA_001741915.1
A1	34.6	50.1	319	–	–	–	Soil	GCA_001741885.1
MOD1-FUNGI2	33.8	50.4	3199	–	–	–	Red seedless grapes	GCA_004634315.1
RAF 106	35.1	49.1	10	–	–	–	Pu-er tea	GCA_011316255.1
L14	36.1	49.3	30	11524	296	–	Marine sponge	JADEYF000000000

It is a matter of controversy that some *A.* niger isolates are renowned for biosynthesis of valuable natural products of nutritional, agrochemical, and pharmaceutical interest, while others are reputed to cause the “black mold” disease (Hayden et al., 1994; Ozer and Koycu, 2006) and produce a plethora of mycotoxins (Sanchez et al., 2012). *A. niger* possesses a bulk warehouse of prolific genes, which involve in regulation of primary and secondary metabolisms (Pel et al., 2007). A genome-scale metabolic network for *A. niger* has been established on account of its high efficiency in rational metabolic design and systems biology studies, such as strain improvement and process optimization (Sun et al., 2007; Lu et al., 2017). Numerous *A. niger* strains have been applied in many fields for a long time. For instance, citric acid as one of incredible organic acids in food industry had been produced on a large scale by *A. niger* 100 years ago (Cairns et al., 2018; Li et al., 2020). It is important that *A. niger* is one of the excellent producers of valuable proteases, which had been widely used as detergents and food ingredients and additives, such as acetylesterase, amylase, fucosidase, glucose oxidase, glucosidase, mannanase, phospholipase, phytase, prolyl endopeptidase, triacylglycerol lipase, trehalase, and xylanase. In addition, numerous chemical studies have indicated that *A. niger* is one of the rich sources of bioactive SMs, with great potential application in agriculture and medicine. Moreover, endoxylanase isozymes of *A. niger* have great potential transforming lignocellulose in pulp and paper industry as industrial bleaching aids (Duarte and Costaferreira, 1994). Furthermore, *A. niger* is also able to deal with the phenolic contaminants in waste water of fermentation broth from industry (Duarte and Costaferreira, 1994). Since genetic engineering is inefficient for fully exploiting in the filamentous fungi industry, a CRISPR (clustered regularly interspaced short palindromic repeats)–Cas9 system had been developed (Nødvig et al., 2015; Nødvig et al., 2018). Based on these genome-editing toolbox, gene inactivation and knockout, gene insertion, base editing, promoter replacement, and regulation of gene expression in *A. niger* have come true. In the future, more importance may be focused on traceless gene editing, multiple gene editing and fine regulation of gene expression in *A. niger*.

## Secondary Metabolites From *Aspergillus niger*

By extensive search on the database of Dictionary of Natural Products (DNP), as many as 166 *A. niger*–derived secondary metabolites (**1**–**166**) were detected till 2020. On the basis of chemical structures, these chemicals are grouped into five types: pyranone, alkaloid, cyclopentapeptide, polyketide, and sterol and, respectively, introduced as follows. (More detailed information about these substances is provided in the Supplementary Materials (Supplementary Table S3).)

### Pyranones

#### *γ*-Naphthylpyradone Monomers

Pyranone derivatives are the most isolated SMs from *A. niger*, including γ-naphthylpyradones (**1**–**31**), α-pyranones (**32**–**56**), and γ-pyranones (**57**–**60**). *A. niger*–derived naphthylpyradones are sorted into two classifications: monomers and dimers, with linear and angular naphtho-γ-pyrone. Fonsecin (**1**) is one of the most frequently isolated γ-naphthylpyradone produced by several *A. niger* strains from various sources, including terrestrial soil (Sakurai et al., 2002), marine (Leutou et al., 2016; Zhou et al., 2016), and plants (Bouras et al., 2005; Fernand et al., 2017; Akinfala et al., 2020). Biological tests suggested that compound 1 possesses dose-dependent inhibitory effect on the interleukin-4 (IL-4) signal transduction and stronger radical scavenging activity against 2,2-diphenyl-1-picrylhydrazyl (DPPH) than ascorbic acid (Sakurai et al., 2002; Leutou et al., 2016). Two analogs TMC-256A1 (**3**) and TMC-256C1 (**8**) also effectively inhibited the IL-4 driven luciferase (Sakurai et al., 2002). However, fonsecin B (**2**) and nigerasperone A (**4**) exhibited weak bioactivity against luciferase and DPPH (Sakurai et al., 2002; Zhang et al., 2007b). One new cytotoxic and antimicrobial rubrofusarin B (**5**) was purified from strain IFB-E003 endophytic on *Cynodon dactylon* Linn. (Song et al., 2004). When cultivated in NaBr or CaBr_2_-containing medium, one marine-derived strain MSA773 was found to secrete a new brominated derivative 6,9-dibromoflavasperone (**9**) with potent radical scavenging activity (Leutou et al., 2016).

**Figure:**
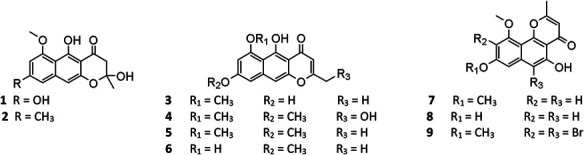


#### *γ*-Naphthylpyradone Dimers

*A. niger*–derived dimeric naphthylpyradones (**10**–**31**) consist of two monomers with linear and/or angular structure(s). It is interesting that most of these bis-naphtho-γ-pyrones were produced by symbiotic *A. niger* strains. Chemical investigation of eight *A. niger* strains led to isolation of the same SM aurasperone A (**10**) (Tanaka et al., 1966; Tanaka et al., 1972; Akiyama et al., 2003; Zhang et al., 2007b; Fang et al., 2016; Li et al., 2016; Wang et al., 2018; Padhi et al., 2020), which possessed a broad spectrum of bioactivities including moderate cytotoxicity (Fang et al., 2016; Padhi et al., 2020), strong antimicrobial effect (Lu et al., 2014; Padhi et al., 2020), and xanthine oxidase (XO) inhibitory and anti-hyperuricosuric activity (Song et al., 2004). Aurasperone B (**15**) had potent radical scavenging activity against DPPH with an IC_50_ value of 0.01 μM (Leutou et al., 2016). Marine strain SCSIO Jcsw6F30 was a prolific producer of asperpyrone-type bis-naphtho-γ-pyrones (BNPs) **10**, **13**–**16**, **18**, **20**–**22**, **24**, and **27**, among which compounds **13**, **16**, and **20** exhibited remarkable inhibitory effects on COX-2 (Fang et al., 2016). In addition to nigerasperone A (**4**), two dimeric naphthylpyradones nigerasperones B (**29**) and C (**19**) were obtained from strain EN-13 and shown to exhibit a moderate radical scavenging effect on DPPH (Zhang et al., 2007b). Bioassay-guided fractionation of the crude extract of strain AKRN associated with *Entandrophragma congoënse* afforded a new antibacterial naphtho-γ-pyrone dimer 2-hydroxydihydronigerone (**30**) (Happi et al., 2015).

**FIGURE 3:**
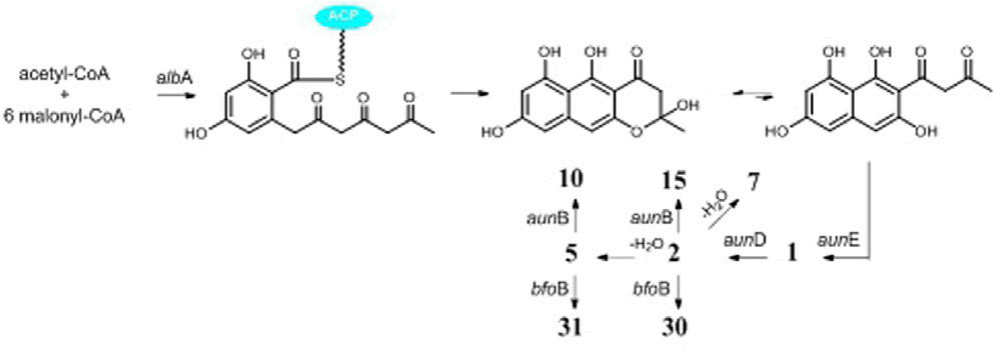
Proposed biosynthetic pathway of γ-naphthylpyridones.

One possible pathway for biosynthesis of γ-naphthylpyradone derivatives had been first proposed by Obermaier and Muller (2019). As shown in Figure 3, one acetyl-CoA and six malonyl-CoA clusters were used as substrates for the biosynthesis of compounds **1**–**3** and 8 by successive catalytic reactions in a nonreducing PKS (nrPKS) system. Two of these monomers further dimerized at various carbon positions (C-6, C-7, C-9, or C-10) and resulted in the formation of dimers **16**, **21**, **27**, and **28**. Lately, one nrPKS gene *D8.t287* responsible for the biosynthesis of the initial precursor heptaketone was identified and characterized by target gene knockout experiment and UPLC-MS analysis (Hua et al., 2020). However, the role of the gene *AunB* or *BfoB* is not confirmed so far.

**Figure:**
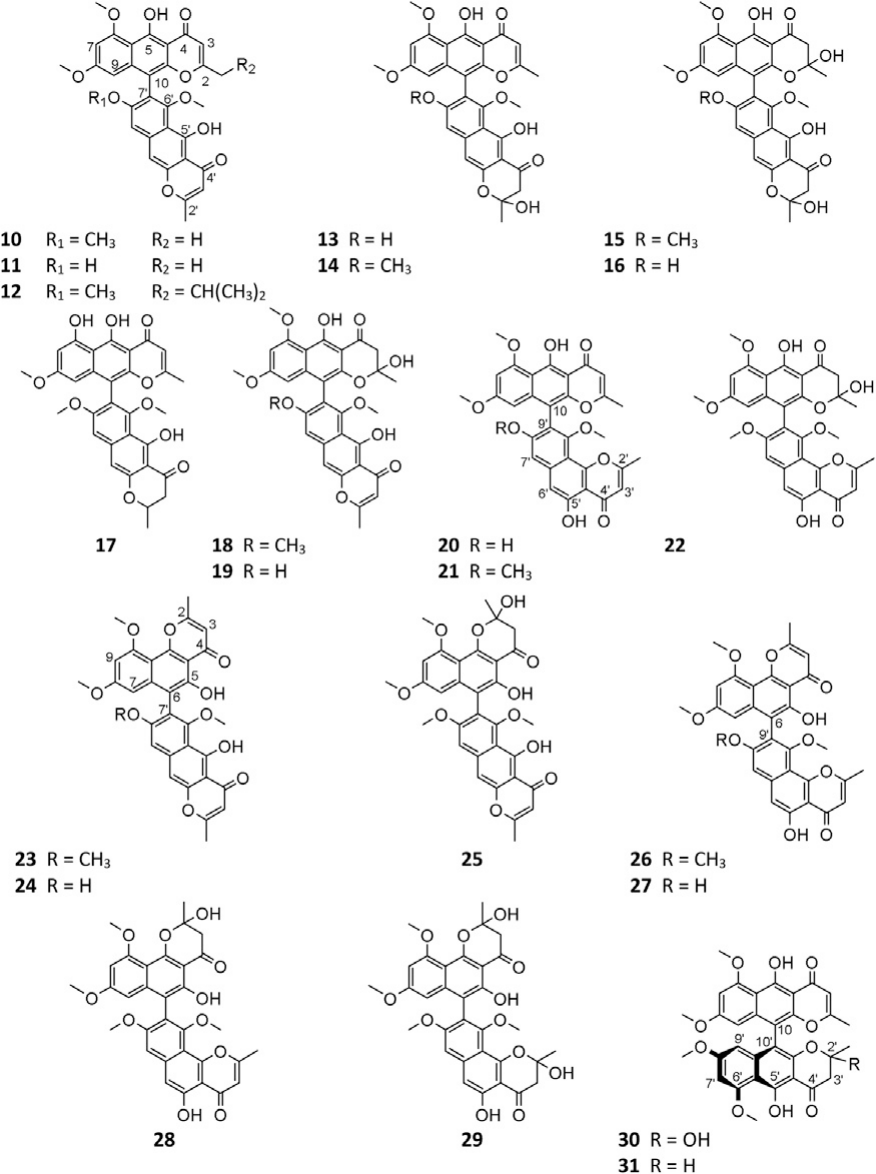


#### *α*-Pyranones

**FIGURE 4:**
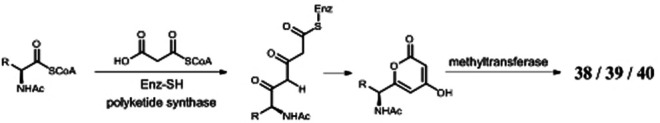
Proposed biosynthetic pathway of campyrones.

**FIGURE 5:**
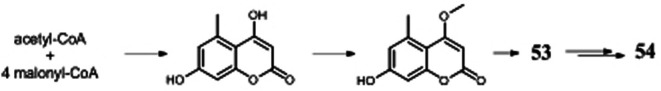
Proposed biosynthetic pathway of orlandin (**53**) and kotanin (**54**).

*A. niger*–derived α-pyranones contain 14 monocyclic compounds (**32**–**40**, **50**), 7 dicyclics (**41**–**47**, **51**), three tricyclics (**48**, **49**, and **56**), and four tetracyclics (**52**–**55**). Chemical analysis of an endophytic *A. niger* strain colonizing in liverwort *Heteroscyphus tener* (Steph.) Schiffn resulted in isolation of three new amide campyrones A–C (**38**–**40**) together with compounds **33** and **34** (Talontsi et al., 2013; Li et al., 2015). One possible biosynthetic pathway proposed by Reber and Burdge (2018) suggested that compounds **38**–**40** were, respectively, formed by one malonyl-CoA and three *N*-acetyl aliphatic amino acids including *L*-valine, *L*-leucine, and *L*-isoleucine (Figure 4), along with two congeners asnipyrones A (**42**) and B (**46**) and nigerapyrones A-H (**35**–**37**, **43**–**45**, **48**–**49**) were first discovered from a mangrove plant–derived strain MA-132 (Liu et al., 2011). Unfortunately, none of these compounds showed potent cytotoxic or antimicrobial activities. Nafuredin (**50**) and bicoumanigrin (**52**) were new α-pyranone analogs produced by marine sponge-derived *A. niger* strains; the former exhibited a powerful and selective inhibitory effect on NFRD (NADH-fumarate reductase) (Takano et al., 2001; Ui et al., 2001) and the latter 3,3′-bicoumarin had moderate cytotoxicity against leukemia and carcinoma cell lines (Hiort et al., 2004). Three 8,8′-bicoumarins, orlandin (**53**), kotanin (**54**), and 7-desmethyl-kotanin (**55**) were produced by a number of *A. niger* strains from various sources, and **53** showed potent inhibitory activity against wheat coleoptile growth at 1 mM but not toxic to day-old cockerels (Cutler et al., 1979; Ovenden et al., 2004; Sorensen et al., 2009; Jomori et al., 2020). Biosynthetically, one acetyl-CoA and four malonyl-CoAs comprised one coumarin through several successive reactions catalyzed by PKSs, followed by formation of compounds 52–55 through dimerization (Figure 5) (Huttel et al., 2003; Huttel and Muller, 2007; Girol et al., 2012). In this pathway, PKS gene *ktnS* was responsible for origination of dimeric coumarins **52**–**55**, gene *ktnB* encode O-methyltransferase, and gene *ktnC* encode CYP450 monooxygenase, manipulating the dimerization of **52**–**55**.

**Figure:**
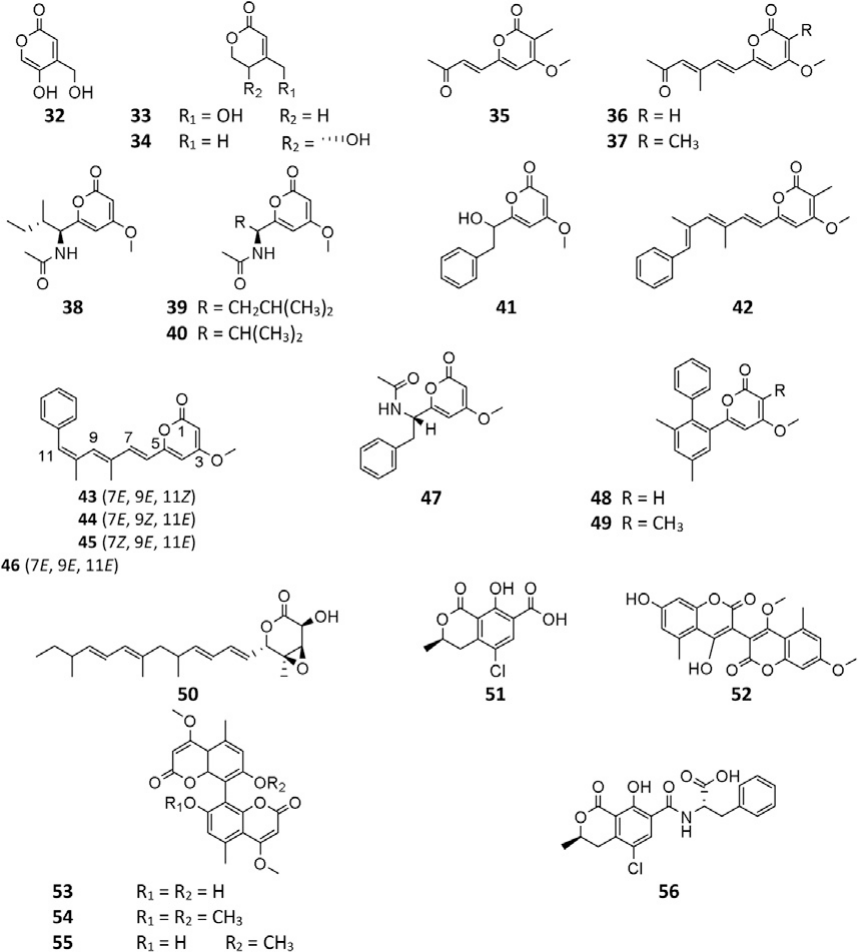


#### *γ*-Pyranones

To the best of our knowledge, only four γ-pyranone derivatives (**57**–**60**) had been detected in SM of *A. niger*. Among these substances, kojic acid (**57**) is the most common product with weak antimicrobial property (Liu et al., 2011; Happi et al., 2015; Padhi et al., 2020). In addition to carbonarone A (**59**) and tensidol B (**60**), one new benzyl γ-pyranone nigerpyrone (**58**) was discovered from a mutant strain FGSC A1279 ΔgcnE and was found to have potent and selective activity against *Candida parapsilosis* (Wang et al., 2018; Padhi et al., 2020).

**Figure:**
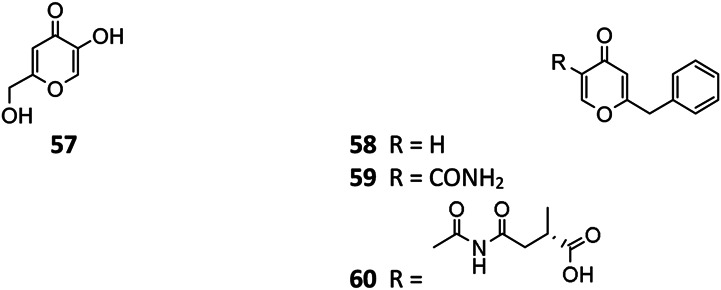


### Alkaloids

#### Pyrroles

**FIGURE 6:**
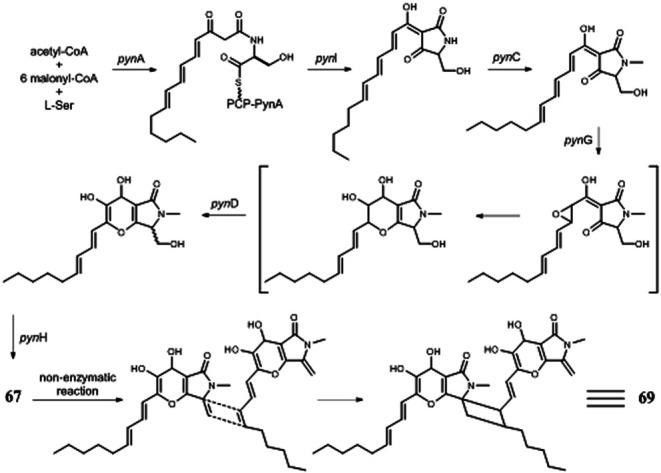
Proposed biosynthetic pathway of pyranonigrin E (**67**) and pyranonigrin F (**69**).

Pyranonigrin derivatives (**61**–**69**) are a family characterized by pyrano [2,3-b] pyrrole skeleton, and their biosynthesis are manipulated by the *pyn* gene cluster in *A. niger* (Riko et al., 2014; Yamamoto et al., 2015). Chemical investigation of one marine sponge–derived strain afforded four pyranonigrins B-D (**61**, **62**, **64**) and Ab (**63**), which 63 showed a strong inhibitory effect on the growth of neonate larvae of the plant pest insect *Spodoptera littoralis* (Hiort et al., 2004). Pyranonigrins A (**65**), S (**66**), and E (**67**) were important agents with potent radical scavenging activity toward DPPH and superoxide (Miyake et al., 2007; Riko et al., 2014). One possible biosynthetic pathway of pyranonigrin E (**67**) had been first proposed by Yamamoto et al. (2015) and coworker in 2015, in which the start units contained one acetyl-CoA, six malonyl-CoAs, and one *L*-Ser (Figure 6), under the action of gene *pynA* (PKS-NRPS hybrid synthase), *pynI* (encode thioesterase), *pynC* (encode methyltransferase), *pynG* (encode flavin-dependent oxidase), *pynD* (encode CYP450), and *pynH* (encode aspartyl protease). After non-enzymatic reaction, two pyranonigrin E (**67**) units could be dimerized to form pyranonigrin F (**69**). One soil-derived *A. niger* strain was found to produce a new dichlorinated pyrrole pyoluteorin (**70**), which obviously induced cell cycle arrest and apoptosis in human triple-negative breast cancer cells MDA-MB-231 (Ding et al., 2020). Two benzyl furopyrrols tensidols A (**71**) and B (**72**) from strain FKI-2342 were potentiators of antifungal miconazole activity (Fukuda et al., 2006) and lately corrected as compounds **59** and **60** (Henrikson et al., 2011).

**Figure:**
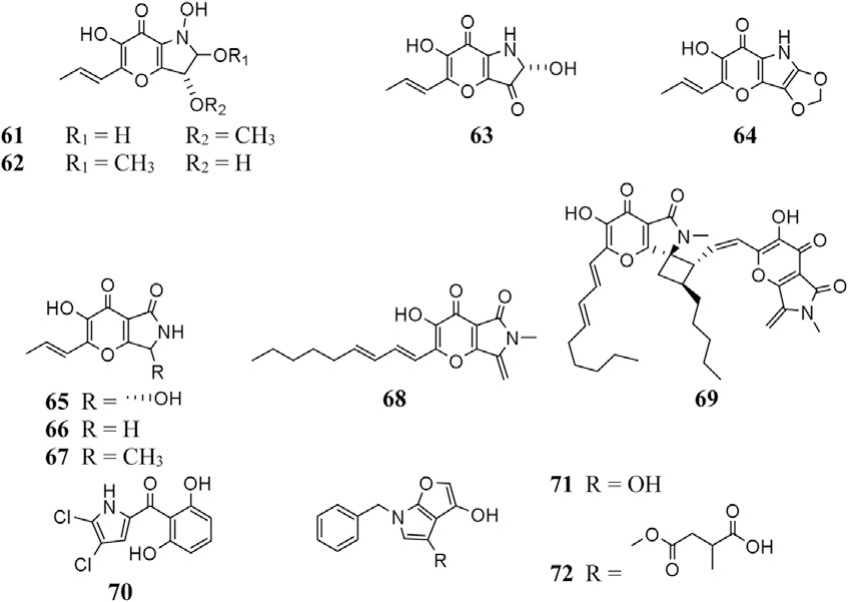


#### Pyridones

*A. niger*–derived pyridone derivatives (**73**–**82**) have one benzyl group and possess antimicrobial and cytotoxic properties (chatr 4). Two new α-pyridones aspernigrins A (**73**) and B_b_ (**74**) were isolated from one *A. niger* strain of marine sponge *Axinella damicornis* and showed moderate cytotoxicity and a potent neuroprotective effect, respectively, (Hiort et al., 2004). When cultivated in fermentation medium containing suberoylanilide hydroxamic acid (SAHA) and *p*-fluoro SAHA, strain ATCC 1015 was discovered to produce three antifungal *γ*-pyridones, nygerones A (**78**), B (**75**), and *p*-fluoro nygerone B (**77**) (Henrikson et al., 2009; Henrikson et al., 2011). In addition to three γ-naphthylpyradones (**1**, **3**, and **5**) and one cyclic peptide (**111**), three 2-benzyl-γ-pyridones aspernigrins B-D (**80**–**82**) were obtained from the marine strain SCSIO Jcsw6F30, and **81** was found to have potent inhibitory activity toward HIV-1 SF162-infected TZM-bl cells (Zhou et al., 2016).

**Figure:**
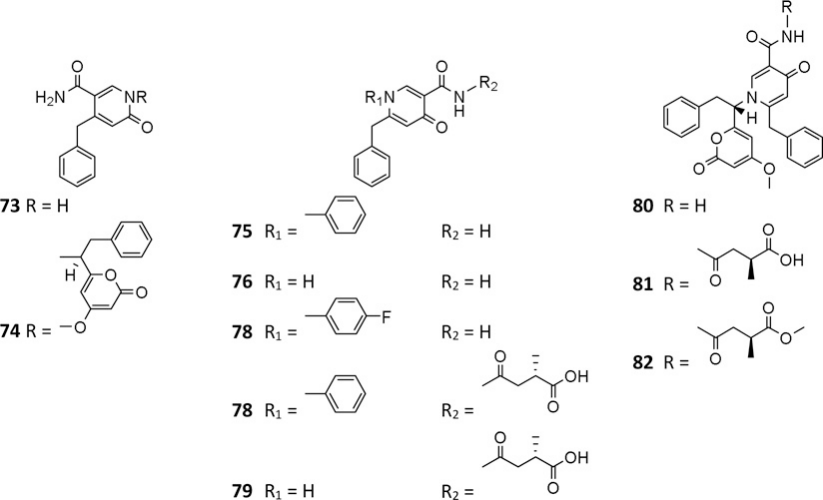


#### Other Alkaloids

Three fatty amines fumonisins B_2_ (**83**), B_1_ (**84**), and B_4_ (**85**) from stains FGSC A1279 and IBT 28144 were carcinogenic (Nielsen et al., 2009; Sorensen et al., 2009; Li et al., 2019). The *aza* gene cluster in strain ATC C1015 was found be responsible for biosynthesis of azanigerone D (**86**) (Zabala et al., 2012). In addition to pyoluteorin (**70**), phenazine-1-carboxylic acid (**87**) was produced by the soil *A. niger* strain (Ding et al., 2020). Two new piperazines nigragillin (**88**) and nigerazine B (**89**) were purified from strain ATCC 11414, and their biosynthesis were regulated by the naphthopyrone precursor BGC *alb* gene cluster (Chiang et al., 2011). Endophytic strain IFB-E003–derived aspernigerin (**90**) displayed a potent effect on the tumor cell lines nasopharyngeal epidermoid KB, cervical carcinoma Hela, and colorectal carcinoma SW1116 (Shen et al., 2006).

**Figure:**
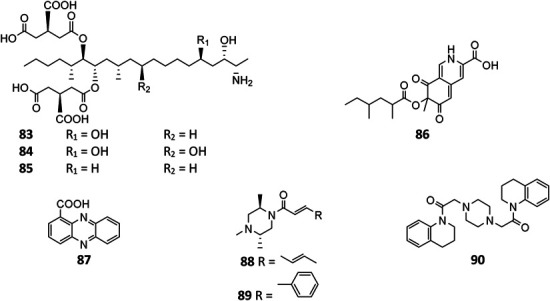


### Amides

Till the end of 2020, only six amides (**91**–**96**) had been isolated and characterized from *A. niger* strains. Fractionation of crude extract of marine strain BRF-074A afforded one furan ester derivative (**91**), one cerebroside chrysogeside D (**93**), and two spiro amides pseurotins A (**95**) and D (**96**), among which 91 exerted a cytotoxic effect on HCT-116 cell line (Uchoa et al., 2017). When cultivated on wheat bran, strains CFR-W-105 and MTCC-5166 were discovered to produce nigerloxin (**92**) with free radical DPPH scavenging activity and inhibitory effect on lipoxygenase-I (LOX-1) and rat lens aldose reductase (RLAR) (Rao et al., 2002; Chakradhar et al., 2009). Ergosterimide (**94**) was a new natural Diels–Alder adduct of ergosteroid and maleimide produced by the strain EN-13 from marine alga (Zhang et al., 2007a).

**Figure:**
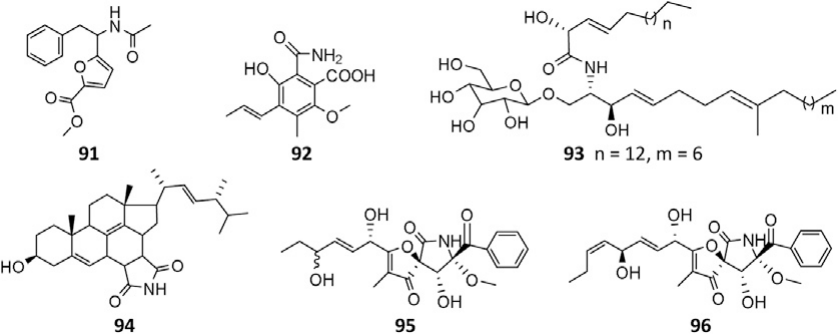


### Cyclopeptides

All peptides of *A. niger* are cyclic and consist of ten dipeptides (**97**–**106**), eight pentapeptides (**107**–**114**), and three bis(dipeptide)s (**115**–**117**). In addition to α-pyranones **32**–**34**, **38**, and **40**, four diketopiperazines (**97**, **99**, **115**, and **116**) were isolated from an endophytic strain of liverwort *Heteroscyphus tener* (Steph.). Schiffn, and compounds **115** and **116** showed weak activity against the human ovarian carcinoma cancer cell line A2780 (Li et al., 2015). However, **115** exhibited significant selective cytotoxicity to human leukemia murine colon 38 and human colon H116 and CX1 cell lines (Varoglu et al., 1997; Varoglu and Crews, 2000). One strain BRF-074A from Northeast Brazilian coast was a prolific producer of cyclopeptides (**101**–**107**, **114**) (Uchoa et al., 2017). Phytochemcial analysis of an uncoded marine strain afforded a new diketopiperazine dimer (**117**) and nine monomers (**98**–**106**) (Ovenden et al., 2004; Zhang et al., 2010; Uchoa et al., 2017). Compounds **98** and **99** had been reported to regulate plant growth (Kimura et al., 1996; Kimura et al., 2005), and **101** had selectively potential cytotoxicity (Graz et al., 2000). Eight malformin analogs (**107**–**114**) were a group of SMs containing structural skeleton of cyclo-*D*-cysteinyl-*D*-cysteinyl-*L*-amino acid-*D*-amino acid-*L*-amino acid (Kim et al., 1993). Malformin A (**107**) demonstrated antibacterial (Suda and Curtis, 1966; Liu et al., 2013) and anticancer activities (Wang et al., 2015), while malformin C (**114**) exhibited a broad spectrum of biological properties including anti-HIV-1 (Zhou et al., 2016), cytotoxic (Jomori et al., 2020), anticancer (Wang et al., 2015), and antibacterial (Suda and Curtis, 1966; Liu et al., 2013).

**Figure:**
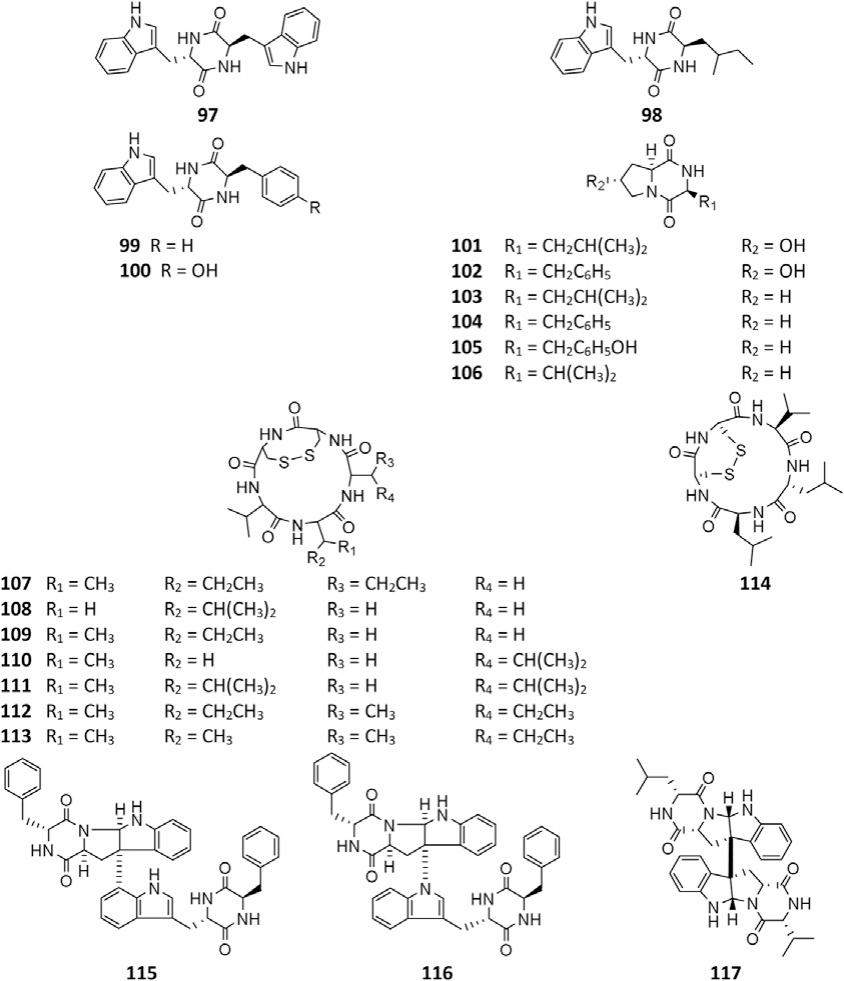


### Polyketides

**FIGURE 7:**
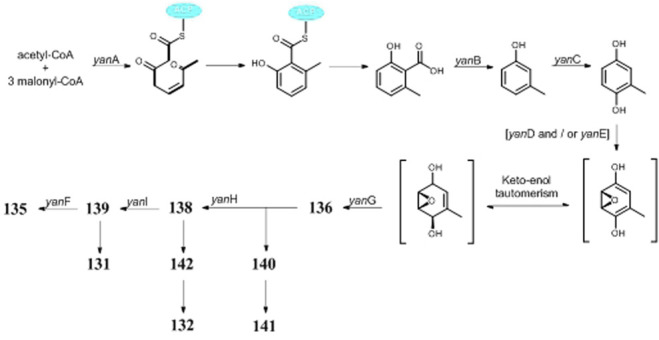
Proposed biosynthetic pathway of yanuthones.

Polyketides (**118**–**155**) are the largest group of SMs produced by *A. niger*. Citric acid (**118**) and itaconic acid (**119**) have been large-scale products in food and pharmaceutical industry for decades (Andersen et al., 2011; Li et al., 2012). Some other valuable chemicals with low molecular weight are also produced by *A. niger*, such as 2-phenylethanol (**128**) (Etschmann et al., 2015), *p*-hydroxyphenylacetic acid (**129**) (Happi et al., 2015), gallic acid (**130**) (Saeed et al., 2020), benzoic acid derivative (**131**) (Zabala et al., 2012), and asperyellone (**147**) (Jefferson, 1967; Chidananda et al., 2008). In comparison with **119**, the biological activity of hexylitaconic acid (**120**) dramatically attenuated (Varoglu et al., 1997; Varoglu and Crews, 2000). By overexpression of transcriptional regulator pBARAGA-CaaR of BGC *caa* in glucose minimal medium, strain ATC C1015 successfully produced three acyltetronic acid derivatives carlosic acid (**123**), carlosic acid methyl ester (**124**), and agglomerin F (**125**) (Yang et al., 2014). Chemical analysis of two strains KB1001 and F97S11 afforded fifteen meroterpenoid derivatives (**132**–**146**), in which biosynthesis was deduced to be manipulated by the *yan* gene cluster in strain KB1001 (Figure 7) (Bugni et al., 2000; Holm et al., 2014). Furthermore, *yan* gene cluster consisted of gene *yanA* [encode 6-methylsalicylic acid synthase (6-MSAS)] together with eight additional genes *yanB* (encode decarboxylase), *yanC* (encode CYP450), *yanD* (encode dehydrogenase), *yanE* (unknown), *yanF* (encode oxidase), *yanI* (encode O-mevalon tiransferase), *yanH* (encode CYP450), and *yanG* (encode prenyl transferase).

**FIGURE 8:**
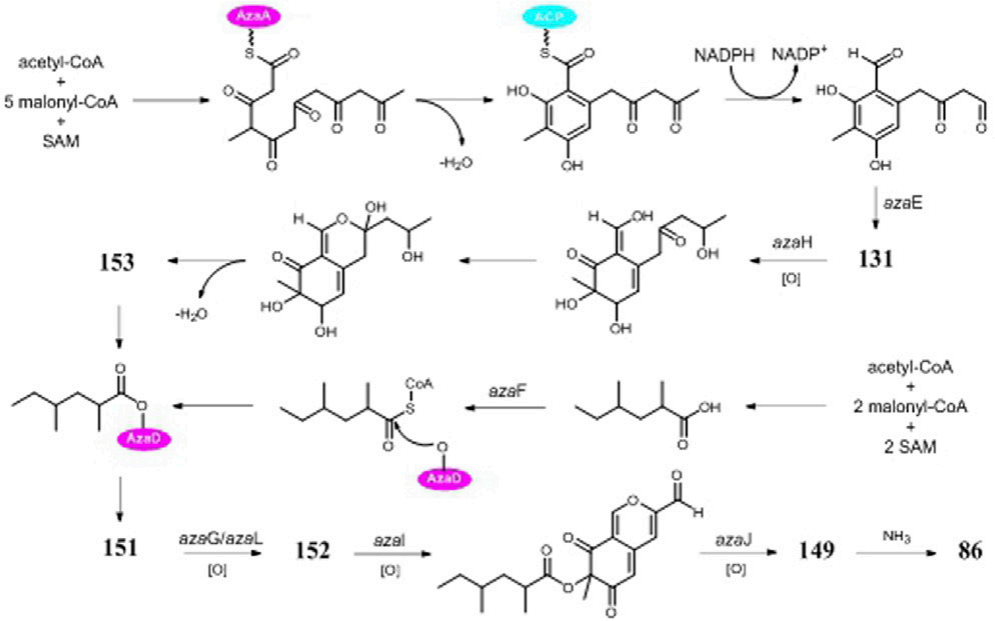
Proposed biosynthetic pathway of pyranoquinones in strain ATCC 1015.

Asperyellone (**147**) was the common product of strains NRRL-3 and CFTRI 1105 (Jefferson, 1967; Chidananda et al., 2008) and exhibited inhibitory effect on lipoxygenase and human platelet aggregation (Rao et al., 2002), UVB protection (Santhakumaran et al., 2019), and antifungal activity (Ayer et al., 1996). In addition to γ-pyridone (**86**), five highly oxygenated pyranoquinones (**86, 149, and 151**–**154**) were detected in SMs of strain T1 by activation of the *aza* gene cluster (Zabala et al., 2012) (Figure 8). In biosynthesis of pyranoquinones, genes *azaE* (encode ketoreductase), *azaF* (encode acyl:CoA ligase), *azaG* (encode FAD-dependent oxygenase), *azaH* (encode salicylate monooxygenase), *azaI* (encode CYP450), *azaJ* (encode dehydrogenase), and *azaL* (encode FAD-dependent oxygenase) play important roles. Funalenone (**150**), one phenalene derivative, was obtained from strain ATCC 11414 whether the *alb*A gene was auxotrophic or not (Chiang et al., 2011). Meanwhile, funalenone (**150**) was also found in *A. niger* mutant Δ*gcn*E (strain FGSC A1279 lacking epigenetic regulator*gcn*E) (Wang et al., 2018). Two tetracycline analogs BMS-192548 (**157**) and TAN-1612 (**158**) were, respectively, obtained from strains WB2346 and ATC C1015 and shown to be acyclic binding inhibitors of neuropeptide Y receptors (Kodukula et al., 1995; Shu et al., 1995; Li et al., 2011).

**Figure:**
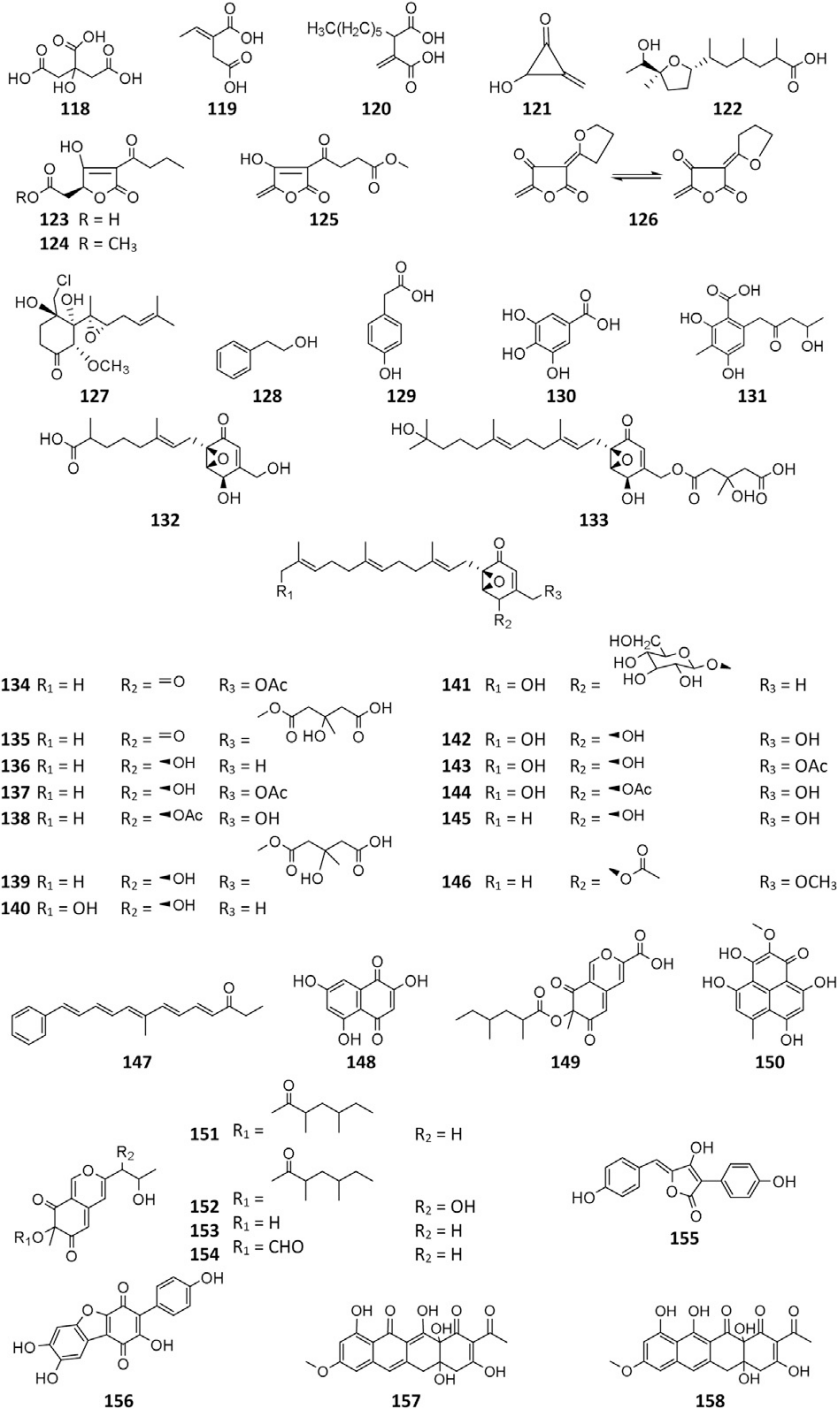


### Sterols

As the by-product of manufacture of citric acid, 14-dehydroergosterol (**159**) and its benzoate (**160**) were the first steroids isolated from *A. niger* (Barton and Bruun, 1951) and possessed anti-inflammatory and cytotoxic properties (Ano et al., 2017). Strain MA-132–derived nigerasterols A (**161**) and B (**162**) had potent antiproliferative activity against human promyelocytic leukemia (HL60) and human lung carcinoma (A549), with IC_50_ values of 0.11 and 0.43 μM, respectively, (Liu et al., 2013). In addition to ergosterimide (**94**), four steroid derivatives (**163**–**166**) were discovered from the endophytic strain EN-13 associated with marine brown alga (Zhang et al., 2007a).

**Figure:**
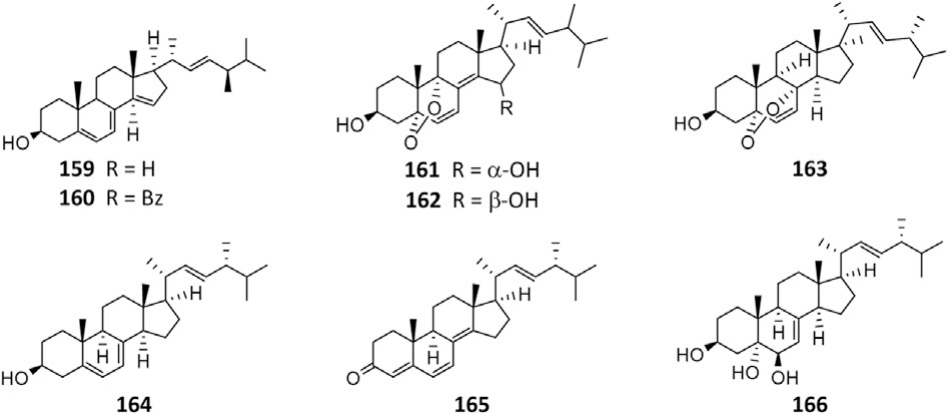


## Conclusion and Future Prospects

*A. niger* strains are ubiquitous in nature and occupy a wide spectrum of habitats in animal and plant environments, and they are economically important both as harmful or beneficial microorganisms. Numerous chemical studies suggest that *A. niger* is one of the prolific sources of functional biomolecules, including organic acids, vitamins, pesticides, valuable proteases, and therapeutic agents, which have potential application in various fields including agriculture, food industry, and medicine. However, the number of new bioactive compounds from *A. niger* has been decreasing for the past 5 years. This deteriorating trend will result in a negative impact on discovery and development of new *A. niger*–derived valuable substances, such as new drug leads. Therefore, more efforts should be made to explore more sources for isolation of new *A. niger* strains and to awaken their silent BGCs to manufacture novel functional biomolecules using new strategies, such as one strain many compounds (OSMAC) approach (Hemphill et al., 2017; Pan et al., 2019) and genetic mining combined with metabolic engineering (Zhang et al., 2019; Li et al., 2020; Wei et al., 2021). Moreover, functional genomics should allow for an in-depth understanding of the underlying biosynthetic logic of *A. niger*–derived SMs (He et al., 2018). In order to accelerate development of valuable products from *A. niger*, construction and breeding of robust strains as well as optimization of their cultivation and fermentation processes should be intensively conducted at various levels (Zou et al., 2015; Xu et al., 2019).
