# Supplementary material for: Aspergillus niger as a Secondary Metabolite Factory
Source: Front Chem. 2021 Jul 30;9:701022. doi: 10.3389/fchem.2021.701022 (PMC8362661; doi:10.3389/fchem.2021.701022)
Supplement: Supplementary file 1 [file Table1.DOCX]

Supplementary Material

*Aspergillus niger* as a secondary metabolite factory

Ronglu Yu, Jia Liu, Yi Wang, Hong Wang and Huawei Zhang*

***Correspondence:** hwzhang@zjut.edu.cn (H. Z.)

**Contents**

**FIGURE S**1. Macroscopic and microscopic morphological characteristics of marine-derived strain L14 2

**TABLE S1.** Biosynthetic gene cluster types of secondary metabolites of 12 wild-type *A. niger* strains 3

**TABLE S2.** BGC similarity of known secondary metabolites in 12 wild-type *A. niger* strains 4

**TABLE S3.** Detail information of all secondary metabolites from known *A. niger* strains 8

**REFERENCE** 25


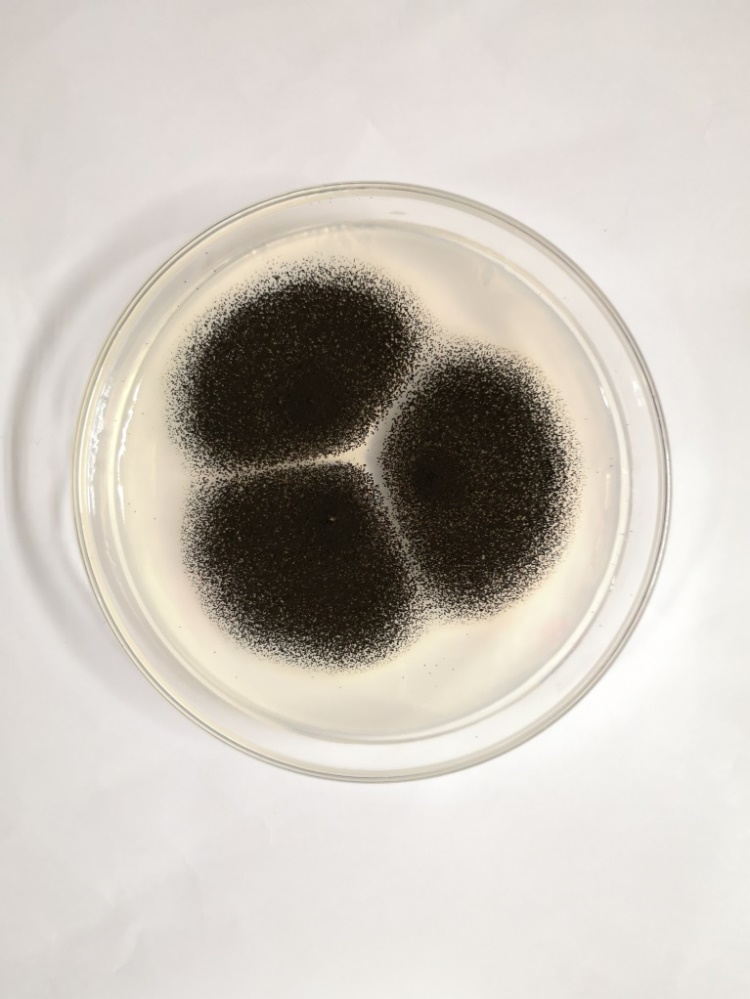

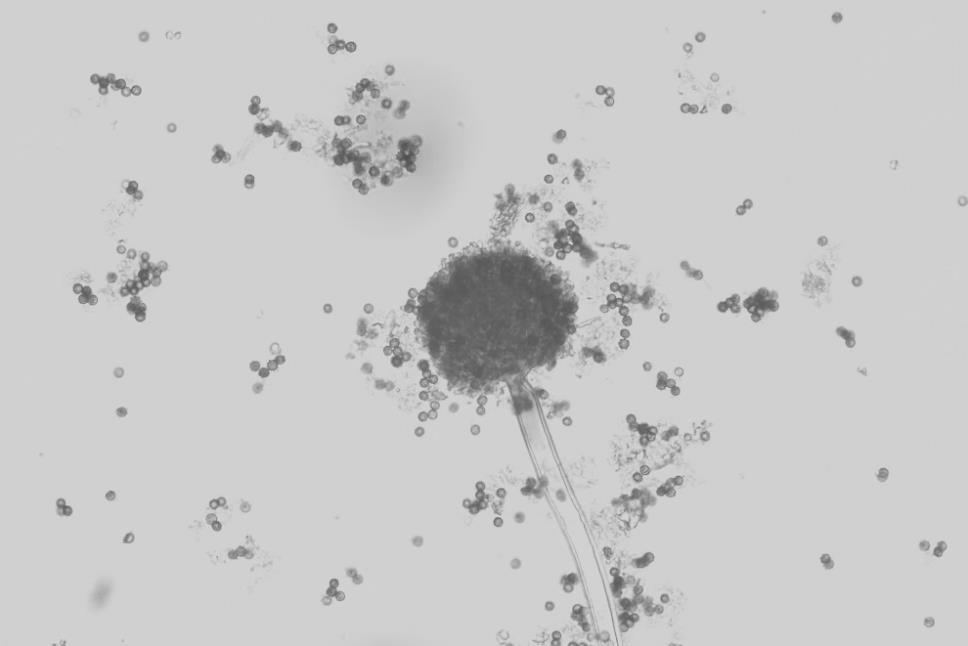


10 × 40

**FIGURE S1.** Macroscopic and microscopic morphological characteristics of marine-derived strain L14

**TABLE S1** Biosynthetic gene cluster (BGC) types of secondary metabolites (SMs) of 12 wild-type *A. niger* strains

| Strains  Quantity  BGC Types | | ATCC 1015 | CBS 513.88 | SH-2 | ATCC 13496 | An76 | JSC-093350089 | H915-1 | L2 | A1 | MOD1-  FUNGI2 | RAF 106 | L14 |
| --- | --- | --- | --- | --- | --- | --- | --- | --- | --- | --- | --- | --- | --- |
| PKS | T1PKS | 12 | 12 | 14 | 14 | 14 | 12 | 13 | 13 | 13 | 16 | 17 | 18 |
|  | T3PKS | 1 | 1 | 1 | 1 | 1 | 1 | 1 | 1 | 1 | 1 | 1 | 1 |
| NRPS | | 18 | 18 | 18 | 18 | 13 | 17 | 18 | 18 | 18 | 22 | 15 | 14 |
| NRPS-like fragment | | 12 | 11 | 12 | 12 | 10 | 12 | 11 | 11 | 10 | 16 | 13 | 13 |
| Hybrid cluster | NRPS + PKS | 8 | 7 | 7 | 8 | 5 | 8 | 8 | 8 | 8 | 6 | 4 | 7 |
|  | NRPS-like + PKS | 4 | 4 | 4 | 4 | 4 | 5 | 4 | 4 | 4 | - | 4 | 4 |
|  | Terpene + PKS | - | - | - | - | - | - | - | - | - | - | 1 | 1 |
|  | Terpene + NRPS | 1 | 1 | 1 | 1 | 2 | - | 1 | 1 | 1 | - | - | 1 |
|  | Terpene + PKS+ NRPS | - | - | - | - | 1 | - | - | - | - | - | 1 | - |
| Terpene | | 9 | 9 | 7 | 8 | 8 | 8 | 8 | 9 | 8 | - | 10 | 8 |
| *β*-lactone containing protease inhibitor | | 1 | 1 | 1 | 1 | 1 | 1 | 1 | 1 | 1 | - | 1 | 1 |
| Indole | | 2 | 2 | 2 | 2 | 2 | 2 | 2 | 2 | 2 | 2 | 2 | 1 |
| Siderophore | | 2 | 2 | 2 | 2 | 2 | 2 | 2 | 2 | 2 | 2 | 1 | - |
| Bacteriocin | | 1 | 1 | 1 | 1 | - | - | 1 | 1 | - | - | - | - |
| Phosphonate | | - | - | - | - | 1 | - | - | - | - | - | - | - |

**TABLE S2** BGC similarity of known secondary metabolites (SMs) in 12 wild-type *A. niger* strains

| BGC types | Strains  Similarity (%)  SM | ATCC 1015 | CBS 513.88 | SH-2 | ATCC 13496 | An76 | JSC-093350089 | H915-1 | L2 | A1 | MOD1-FUNGI2 | RAF 106 | L14 |
| --- | --- | --- | --- | --- | --- | --- | --- | --- | --- | --- | --- | --- | --- |
| Hybrid | AbT1 | 100 | - | 100 | 100 | 100 | 100 | 100 | 100 | 100 | - | 100 | 100 |
|  | ajudazol A | - | - | - | - | - | - | - | - | - | - | 38 | - |
|  | ankaflavin / monascin / rubropunctatine / monascorubrin | - | - | - | - | - | 16 | - | - | - | - | - | - |
|  | azanigerone A | 100 | 100 | 100 | 100 |  | 100 | 100 | 100 | 100 | - | - | - |
|  | burnettramic acid | - | 22 | - | - | - | - | - | - | - | - | - | - |
|  | communesins | 12 | 12 | 12 | 12 | 12 | 12 | 12 | 12 | 12 |  | 12 | - |
|  | cytochalasin E / cytochalasin K |  | - |  |  |  |  |  |  | - | - | - | 15 |
|  | dihydroisoflavipucine / isoflavipucine | 18 | - | 18 | 18 | 18 | 18 | 18 | 18 | 18 | 12 | - | - |
|  | fumonisin | 52 | 52 | - | - | - | - | 52 | 52 | - | - | - | - |
|  | fumonisin B1 | - | - | 37 | 37 |  | 37 | - | - | 41 | 41 | 8 | - |
|  | fusarin | - | 100 | 100 | 100 | - | - | 100 | 100 | 100 | 100 | - | - |
|  | leucinostatin A / leucinostatin B | - | - | - | - | 10 | - | - | - | - | - | - | - |
|  | monacolin K | - | - | - | - | 22 | - | - | - | - | - | - | - |
|  | notoamide A | 16 | 16 | 16 | 16 | 16 | 11 | 16 | 16 | 16 | - | 11 | 11 |
|  | phenalamide A2 | - | - | - | 33 | - |  | - | - | - | - | 25 | - |
|  | phyllostictine A / B | - | - | - | - | - | 30 | 20 | - | - | - | - | 20 |
|  | pyralomicin 1a | 7 | 7 | 7 | - | - | 7 | - | 7 | 7 | - | - | - |
|  | pyranonigrin E | - | - | - | - | - | - | - | - | - | - | - | 100 |
|  | squalestatin S1 | - | - | - | - | - | - | - | - | - | - | 9 | - |
|  |  |  |  |  |  |  |  |  |  |  |  |  |  |
|  | TAN-1612 | - | - | - | - | 100 | - | - | - | - | - | - | - |
|  | xenolozoyenone | 100 | - | - | - | - | 100 | - | - | - | 100 | 100 | - |
|  | ucs1025a | - | - | - | - | 15 | - | - | - | - | - | - | - |
| Indole | notoamide A | - | - | - | - | 11 | - | - | - | 11 | - | - | - |
| NRPS | AbT1 | 100 | 100 | - | 100 | 100 | - | - | 100 | 100 | 100 | - | 100 |
|  | ajudazol A | - | - | - | - | - | - | 53 | 53 | - | - | 53 | - |
|  | aspercryptins | - | - | - | - | 13 | - | - | - | - | - | 13 | - |
|  | aspirochlorine | 9 | 9 | 9 | 9 |  | 9 | - | - | - | 9 | - | - |
|  | bacillomycin D | 20 | - | - | - | - |  | 20 | - | - | - | - | - |
|  | chromane | 33 |  |  |  |  |  |  |  |  |  |  |  |
|  | curacin A | - | 28 | 28 | 46 | - |  | - | - | 26 | - | - | - |
|  | ferrichrome | 66 | 66 | 66 | 66 | - | 66 | 66 | 66 | 66 | 66 | - | - |
|  | iturin | - | 22 | - | 22 | 22 | 22 | - | 22 | - | - | 22 | - |
|  | monacolin K | 22 | 100 | 22 | 22 | - | 22 | 22 | 22 | 22 | - | - | - |
|  | nidulanin A | 100 | 100 | 100 | 100 | 100 | 100 | 100 | 100 | - | - | 100 | 50 |
|  | nodulisporic acid F | 15 | 15 | 15 | 15 | - | 15 | 15 | 15 | 15 | - | - | - |
|  | novofumigatonin | - | - | - | - | - | - | - | - | - | - | - | 15 |
|  | ochratoxin A | - | 100 | 100 | 100 | - | - | 100 | 100 | 80 | - | - | - |
|  | phyllostictine A / B | 30 | 30 | 30 | 30 | - | - | - | 30 | 30 | - | - | - |
|  | pyrichalasin H | - | - | - | - | - | 18 | - | - | - | - | - | - |
|  | serinocyclin A / B | - | - | - | - |  | 100 | - | - | - | 100 | - | - |
|  | tryptoquialanine | - | - | - | - | 9 | - | - | - | - | - | - | - |
| NRPS-like | communesins |  | - |  |  |  |  |  |  | - | 12 | - | - |
|  | EQ-4 | - | - | - | - | 66 | - | - | - | - | - | 66 | 66 |
|  | fusaric acid | 13 | 13 | 13 | - | - | 13 | - | - | 13 | - | - | - |
| PKS | 4-epi-15-epi-brefeldin A | - | - | - | - | - | - | - | - | - | - | 20 | - |
|  | aflavarin | 60 | 80 | 80 | 80 |  | 80 | 80 | 80 | 80 | 60 | 40 | - |
|  | ajudazol A | 38 | 38 | 38 | 38 | - | - | 38 | 38 | - | - | - | - |
|  | ankaflavin / monascin / rubropunctatine / monascorubrin | 16 | - | 16 | 16 | 16 | - | 16 | 16 | 16 | - | 16 | - |
|  | azanigerone A |  | - | - | - | - | - | - | - | 100 | 86 | - | 73 |
|  | BE-43547A1 / BE-43547A2 / BE-43547B1 / BE-43547B2 / BE-43547B3 / BE-43547C1 / BE-4 | - | 10 | - | 10 | - | - | 10 | 10 | 10 | - | - | - |
|  | chromane |  | - | - |  | - | 33 | - | - | - | - | - | - |
|  | citreohybridonol | 18 | 18 | 18 | 18 | - | 18 | 18 | 18 | 18 | - | - | - |
|  | citrnin | - | - | 12 | - | - |  | - | - | 12 | - | 18 | - |
|  | duclauxin | 50 | 50 | 57 | 50 | 50 | 50 | 50 | 50 | 50 | 57 | 57 | - |
|  | ebelactone A / B | - | - | - | - | - | 40 | - | - | - | - | - | - |
|  | fumonisin | - | - | - | - | - | - | - | - | - | - | 11 | - |
|  | fumonisin B1 | - | - | - | - | 8 | - | - | - | - | - | - | - |
|  | fusarielin H | - | - | - | - | - | - | - | - | - | - | 25 | - |
|  | gibberellin | - | - | - | - | 28 |  | - | - | - | - | 28 | - |
|  | Lacunalides | - | - | - | - | 19 |  | - | - | - | - | - | - |
|  | leucinostatin A / B | 10 | 10 | 10 | - | 10 | 10 | 10 | 10 | 10 | 10 | 10 | - |
|  | melanin | 100 | 100 | 100 | 100 | 100 | 100 | 100 | 100 | 100 | 100 | 100 | 100 |
|  | nannocystin a | - | - | - | - | 21 | - | - | - | - | - | - | - |
|  | neurosporin A | - | - | - | - | 46 | - | - | - | - | - | 40 | - |
|  | oligomycin | - | - | - | - | - | - | - | - | - | 38 | - | - |
|  | pyranonigrin E | 100 | - | - | - | - | 100 | 100 | 100 | - | - | 100 | 100 |
|  | pyxipyrrolone A / B | - | - | - | 11 | - | - | - | - | - | - | - | - |
|  | quartromicin A1 | - | - | - | - | - | - | - | - | - | 5 | - | - |
|  | rhizopodin | - | 23 | 23 | 23 | - | - | - | - | - | - | - | - |
|  | rosamicin / salinipyrone A / pacificanone A | - | - | - | - | - | - | - | - | 13 | - | - | - |
|  | sorangicin A | - | - | - | - | - | - | - | - | - | - | 17 | - |
|  | spirangien O | - | - | - | - | 33 | - | - | - | - | 26 | - | - |
|  | stigmatellin | - | - | 40 | - | - | - | - | - | - | 35 | - | - |
|  | TAN-1612 | 100 | 100 | 100 | 100 | - | 100 | 100 | 100 | 100 | 60 | 100 | 100 |
|  | tylactone | - | - | - | - | 10 | - | - | - | - | - | - | - |
|  | ucs1025a | - | - | - | - | 15 | 15 | - | - | - | - | - | - |
|  | yanuthone D | 100 | 100 | 100 | 100 | - | 100 | 100 | 100 | 100 | 80 | 100 | 80 |
| Terpene | ACT-Toxin II | - | - | - | - | - | - | - | - | - | - | 100 | - |
|  | clavaric acid | - | - | - | 100 | - |  | - | - | - | - | - | 100 |
|  | squalestatin S1 | 40 | 40 | - | 40 | 40 | - | 40 | - | 40 | 40 | 40 | 40 |

**TABLE S3** Detail information of all secondary metabolites from known *A. niger* strains

1. **Pyranones**

**1.1 *γ*-Naphthylpyradone monomers**

| **Compound NO.** | **Name** | **Strain** | **Source** | **Bioactivity** | **Ref.** |
| --- | --- | --- | --- | --- | --- |
| **1** | fonsecin | SCSIO Jcsw6F30 | Marine alga *Sargassum* sp. | - | 1 |
|  |  | - | Two cocoa (*Theobroma cacao* L.) bean (F and T series) hybrids | - | 2 |
|  |  | TC 1629 | Soil (Tokyo, Japan) | Enzyme inhibitor(IL-4 driven luciferase) | 3 |
|  |  | MSA773 | Marine mudﬂat | Radical scavenging activity(DPPH) | 4 |
|  |  | C-433 | Grapes | - | 5 |
|  |  | - | *Penaeus monodon* | - | 6 |
| **2** | fonsecin B | TC 1629 | Soil(Tokyo, Japan) | - | 3 |
| **3** | TMC-256A1 | 15F41-1-3 | Unidentified marine sponge | - | 7 |
|  |  | - | Marine | - | 8 |
|  |  | TC 1629 | Soil(Tokyo, Japan) | Enzyme inhibitor(IL-4 driven luciferase) | 3 |
|  |  | MSA773 | Marine mudﬂat | Radical scavenging activity(DPPH) | 4 |
| **4** | nigerasperone A | EN-13 | Marine brown alga *Colpomenia sinuosa* | - | 9 |
| **5** | rubrofusarin B | SCSIO Jcsw6F30 | Marine alga *Sargassum* sp. | - | 1 |
|  |  | IFB-E003 | Leaves of *Cynodon dactylon*(L.) | Cytotoxicity(colon cancer cell line SW1116), XO inhibitive activity, antimicrobial activity(*B. subtilis*, *E. coli,* *T. rubrum* etc.) | 10 |
|  |  | TC 1629 | Soil(Tokyo, Japan) | - | 3 |
|  |  | MSA773 | Marine mudﬂat | Radical scavenging activity(DPPH) | 4 |
| **6** | rubrofusarin | SCSIO Jcsw6F30 | Marine alga *Sargassum* sp. | - | 1 |
| **7** | flavasperone | TC 1629 | Soil(Tokyo, Japan) | - | 3 |
|  |  | 2HL-M-8 | Mud(Huludao coastline, China) | - | 11 |
|  |  | MSA773 | Marine mudﬂat | Radical scavenging activity(DPPH) | 4 |
|  |  | - | *Penaeus monodon* | - | 6 |
| **8** | TMC-256C1 | - | Marine | - | 8 |
|  |  | TC 1629 | Soil (Tokyo, Japan) | Enzyme inhibitor(IL-4 driven luciferase) | 3 |
| **9** | 6,9-dibromoﬂavasperone | MSA773 | Marine mudﬂat | Radical scavenging activity(DPPH) | 4 |

**1.2 *γ*-Naphthylpyradone dimers**

| **Compound NO.** | **Name** | **Strain** | **Source** | **Bioactivity** | **Ref.** |
| --- | --- | --- | --- | --- | --- |
| **10** | aurasperone A / isoaurasperone A | EN-13 | Marine brown alga *Colpomenia sinuosa* | Antifungal activity(*C. albicans*), radical scavenging activity(DPPH) | 9 |
|  |  | FGSC A1279 ΔgcnE mutant | - | - | 12 |
|  |  | BL-5-1 | - | - | 13, 14,15 |
|  |  | SCSIO Jcsw6F30 | Marine alga *Sargassum sp.* | Cytotoxicity(K562, A549, Du145 etc.) | 16 |
|  |  | JV-33-48 | Soil(Sakai) | Enzyme inhibitor(*Taq* DNA polymerase) | 17 |
|  |  | Tiegh | Lichen thallus *Parmotrema ravum* | Antimicrobial activity(*P. aeruginosa*, *S. aureus* and *C. krusei*) | 18 |
|  |  | IFB-E003 | Leaves of *Cynodon*  *dactylon* | Cytotoxicity(colon cancer cell line SW1116), XO inhibitive activity, antimicrobial activity(*B. subtilis*, *E. coli,* *T. rubrum* etc.) | 10 |
|  |  | 2HL-M-8 | Mud(Huludao coastline, China) | - | 11 |
|  |  | ATCC 11414 | - | - | 19 |
| **11** | aurasperone D / dianhydroaurasperone C | Tiegh | Lichen thallus *Parmotrema ravum* | - | 18 |
|  |  | EN-13 | Marine brown alga *Colpomenia sinuosa* | - | 9 |
| **12** | aurasperone H | 2HL-M-8 | Mud(Huludao coastline, China) | Cytotoxicity(A549 and HL60 cell lines) | 11 |
| **13** | aurasperone F | SCSIO Jcsw6F30 | Marine alga *Sargassum sp.* | Cytotoxicity(K562, A549, Du145 etc.), enzyme inhibitor(COX-2) | 16 |
|  |  | C-433 | Grapes | - | 5 |
| **14** | fonsecinone D / aurasperone E | C-433 | Grapes | - | 5 |
|  |  | EN-13 | Marine brown alga *Colpomenia sinuosa* | Radical scavenging activity(DPPH) | 9 |
|  |  | 2HL-M-8 | Mud(Huludao coastline, China) | - | 11 |
|  |  | SCSIO Jcsw6F30 | Marine alga *Sargassum sp.* | Cytotoxicity(K562, A549, Du145 etc.) | 16 |
|  |  | FGSC A1279 ΔgcnE mutant | - | - | 12 |
| **15** | aurasperone B | EN-13 | Marine brown alga *Colpomenia sinuosa* | - | 9 |
|  |  | BL-5-1 | - |  | 13, 14, 15 |
|  |  | MSA773 | Marine mudﬂat | Radical scavenging activity(DPPH) | 4 |
|  |  | SCSIO Jcsw6F30 | Marine alga *Sargassum sp.* | Cytotoxicity(K562, A549, Du145 etc.) | 16 |
|  |  | C-433 | Grapes | - | 5 |
|  |  | IBT 28144 (CBS 101705) | - | - | 20 |
|  |  | - | *Penaeus monodon* | - | 6 |
|  |  | ATCC 11414 | *-* | - | 19 |
| **16** | aurasperone C | 15F41-1-3 | Unidentified marine sponge | - | 7 |
|  |  | BL-5-1 | - | - | 13, 14, 15 |
|  |  | SCSIO Jcsw6F30 | Marine alga *Sargassum sp.* | Cytotoxicity(K562, A549, Du145 etc.), enzyme inhibitor(COX-2) | 16 |
|  |  | C-433 | Grapes | - | 5 |
|  |  | - | *Penaeus monodon* | - | 6 |
| **17** | aurasperone D_b_ | C-433 | Grapes | - | 5 |
| **18** | fonsecinone B | EN-13 | Marine brown alga *Colpomenia sinuosa* | - | 9 |
|  |  | 2HL-M-8 | Mud(Huludao coastline, China) | - | 11 |
|  |  | SCSIO Jcsw6F30 | Marine alga *Sargassum sp.* | Cytotoxicity(K562, A549, Du145 etc.) | 16 |
|  |  | ATCC 11414 | - | - | 19 |
| **19** | nigerasperone C | EN-13 | Marine brown alga *Colpomenia sinuosa* | Antifungal activity(*C. albicans*), radical scavenging activity(DPPH) | 9 |
| **20** | asperpyrone A | Tiegh | Lichen thallus *Parmotrema ravum* | - | 18 |
|  |  | EN-13 | Marine brown alga *Colpomenia sinuosa* | - | 9 |
|  |  | SCSIO Jcsw6F30 | Marine alga *Sargassum sp.* | Cytotoxicity(K562, A549, Du145 etc.), enzyme inhibitor(COX-2) | 16 |
|  |  | JV-33-48 | Soil(Sakai) | Enzyme inhibitor(*Taq* DNA polymerase) | 17 |
| **21** | fonsecinone A | Tiegh | Lichen thallus *Parmotrema ravum* | Antimicrobial activity(*S. aureus, E. coli* and *P. syringae*) | 18 |
|  |  | - | Soil | - | 21 |
|  |  | IFB-E003 | Leaves of *Cynodon*  *dactylon* | Antimicrobial activity(*B. subtilis*, *E. coli,* *T. rubrum* etc.); XO inhibitive activity | 10 |
|  |  | EN-13 | Marine brown alga *Colpomenia sinuosa* | Antifungal activity(*C. albicans*) | 9 |
|  |  | 2HL-M-8 | Mud(Huludao coastline, China) | - | 11 |
|  |  | SCSIO Jcsw6F30 | Marine alga *Sargassum sp.* | Cytotoxicity(K562, A549, Du145 etc.) | 16 |
|  |  | JV-33-48 | Soil(Sakai) | Enzyme inhibitor(*Taq* DNA polymerase) | 17 |
|  |  | ATCC 11414 | - | - | 19 |
| **22** | fonsecinone C | EN-13 | Marine brown alga *Colpomenia sinuosa* | - | 9 |
|  |  | 2HL-M-8 | Mud(Huludao coastline, China) | Cytotoxicity(HL60 cell lines) | 11 |
|  |  | SCSIO Jcsw6F30 | Marine alga *Sargassum sp.* | Cytotoxicity(K562, A549, Du145 etc.) | 16 |
|  |  | ATCC 11414 | - | - | 19 |
| **23** | asperpyrone C | EN-13 | Marine brown alga *Colpomenia sinuosa* | Antifungal activity(*C. albicans*) | 9 |
|  |  | 2HL-M-8 | Mud(Huludao coastline, China) | - | 11 |
|  |  | JV-33-48 | Soil(Sakai) | - | 17 |
|  |  | ATCC 11414 | - | - | 19 |
| **24** | asperpyrone D | SCSIO Jcsw6F30 | Marine alga *Sargassum sp.* | Cytotoxicity(K562, A549, Du145 etc.) | 16 |
| **25** | 1356390-59-4***** | ATCC 11414 | - | - | 19 |
| **26** | asperpyrone B | IFB-E003 | Leaves of *Cynodon*  *dactylon* | Antimicrobial activity(*B. subtilis*, *E. coli,* *T. rubrum* etc.) | 10 |
|  |  | 2HL-M-8 | Mud(Huludao coastline, China) | - | 11 |
|  |  | JV-33-48 | Soil(Sakai) | - | 17 |
|  |  | ATCC 11414 | - | - | 19 |
| **27** | asperpyrone E | SCSIO Jcsw6F30 | Marine alga *Sargassum sp.* | Cytotoxicity(K562, A549, Du145 etc.) | 16 |
| **28** | 1356390-58-3***** | ATCC 11414 | - | - | 19 |
| **29** | nigerasperone B | EN-13 | Marine brown alga *Colpomenia sinuosa* | - | 9 |
| **30** | 2-hydroxydihydronigerone | AKRN | Roots of *Entandrophragma congoënse* | Antibacterial activity(*E. aerogenes, E. cloacae, K. pneumonia* etc.) | 22 |
| **31** | nigerone | AKRN | Roots of *Entandrophragma congoënse* | Antibacterial activity(*E. aerogenes, E. cloacae, K. pneumonia* etc.) | 22 |

*****: CAS registry number

**1.3 *α*-Pyranones**

| **Compound NO.** | **Name** | **Strain** | **Source** | **Bioactivity** | **Ref.** |
| --- | --- | --- | --- | --- | --- |
| **32** | 4-(hydroxymethyl)-5-hydroxy-2H-pyran-2-one | AKRN | Roots of *Entandrophragma congoënse* | Antibacterial activity(*E. aerogenes, E. cloacae, K. pneumonia* etc.) | 22 |
| **33** | 4-(hydroxymethyl)5,6-dihydro-pyran-2-one | - | Liverwort *Heteroscyphus tener* (Steph.) Schiffn | - | 23 |
| **34** | walterolactone A | - | Liverwort *Heteroscyphus tener* (Steph.) Schiffn | - | 23 |
| **35** | nigerapyrone E | MA-132 | Marine mangrove plant *Avicennia marina* | Cytotoxicity(SW1990, MDA-MB-231, A549 etc. cell lines) | 24 |
| **36** | nigerapyrone C | MA-132 | Marine mangrove plant *Avicennia marina* | - | 24 |
| **37** | nigerapyrone D | MA-132 | Marine mangrove plant *Avicennia marina* | Cytotoxicity(MCF-7, HepG2 and A549 cell lines) | 24 |
| **38** | campyron A / campyrone A | CAFT160 | *Zanthoxylum lemairei* | - | 25 |
|  |  | - | Liverwort *Heteroscyphus tener* (Steph.) Schiffn | - | 23 |
| **39** | campyrone B | CAFT160 | *Zanthoxylum lemairei* | - | 25 |
|  |  | - | Liverwort *Heteroscyphus tener* (Steph.) Schiffn | - | 23 |
| **40** | campyrone C | CAFT160 | *Zanthoxylum lemairei* | - | 25 |
|  |  | - | Liverwort *Heteroscyphus tener* (Steph.) Schiffn | - | 23 |
| **41** | aspergillusol | EN-13 | Marine brown alga *Colpomenia sinuosa* | - | 26 |
| **42** | asnipyrone A | MA-132 | Marine mangrove plant *Avicennia marina* | Cytotoxicity(A549 cell lines) | 24 |
| **43** | nigerapyrone F | MA-132 | Marine mangrove plant *Avicennia marina* | - | 24 |
| **44** | nigerapyrone G | MA-132 | Marine mangrove plant *Avicennia marina* | - | 24 |
| **45** | nigerapyrone H | MA-132 | Marine mangrove plant *Avicennia marina* | - | 24 |
| **46** | asnipyrone B | MA-132 | Marine mangrove plant *Avicennia marina* | - | 24 |
| **47** | pyrophen | EN-13 | Marine brown alga *Colpomenia sinuosa* | - | 26 |
|  |  | Tiegh | Lichen thallus *Parmotrema ravum* | Antimicrobial activity(*C. krusei, C. parapsilosis, M. luteus* etc.) | 18 |
|  |  | 94-1212 | *Hyrtios proteus* sponge | - | 27, 28 |
|  |  | AKRN | Roots of *Entandrophragma congoënse* | Antibacterial activity(*E. aerogenes, E. cloacae, K. pneumonia* etc.) | 22 |
| **48** | nigerapyrone A | MA-132 | Marine mangrove plant *Avicennia marina* | - | 24 |
| **49** | nigerapyrone B | MA-132 | Marine mangrove plant *Avicennia marina* | Cytotoxicity(HepG2 cell lines) | 24 |
| **50** | nafuredin | FT-0554 | Marine sponge | NADH-fumarate reductase (NFRD) inhibitor | 29, 30 |
| **51** | ochratoxin alpha | IBT 28144 (CBS 101705) | - | - | 20 |
| **52** | bicoumanigrin | - | Mediterranean sponge *Axinella damicornis* | Antiproliferative activity(leukemia and carcinoma cell lines) | 31 |
| **53** | orlandin | FGSC A1180 | - | - | 32 |
|  |  | ATCC 36626 | Orange leaves | Plant Growth Inhibitor(wheat coleoptile) | 33 |
|  |  | IBT 28144 (CBS 101705) | - | - | 20 |
| **54** | kotanin | FGSC A1180 | - | - | 32 |
|  |  | - | - | Day-Old Chicks toxic activity | 33 |
|  |  | IBT 28144 (CBS 101705) | - | - | 20 |
|  |  | ATCC 11414 | - | - | 19 |
| **55** | desmethylkotanin / 7-demethylkotanin | FGSC A1180 | - | - | 32 |
|  |  | - | Marine derived | - | 8 |
|  |  | 15F41-1-3 | Unidentified marine sponge | - | 7 |
|  |  | IBT 28144 (CBS 101705) | - | - | 20 |
| **56** | ochratoxin A | IBT 28144 (CBS 101705) | - | - | 20 |
|  |  | - | Brazilian cocoa beans | - | 34 |

**1.4 *γ*-Pyranones**

| **Compound NO.** | **Name** | **Strain** | **Source** | **Bioactivity** | **Ref.** |
| --- | --- | --- | --- | --- | --- |
| **57** | kojic acid | FGSC A1279 | - | - | 35 |
|  |  | AKRN | Roots of *Entandrophragma congoënse* | Antibacterial activity(*E. aerogenes, E. cloacae, K. pneumonia* etc.) | 22 |
| **58** | aspergyllone / nigerpyrone | Tiegh | Lichen thallus *Parmotrema ravum* | Antifungal activity(*C. parapsilosis*) | 18 |
|  |  | FGSC A1279 ΔgcnE mutant | - | - | 12 |
| **59** | carbonarone A | Tiegh | Lichen thallus *Parmotrema ravum* | - | 18 |
|  |  | FGSC A1279 ΔgcnE mutant | - | - | 12 |
|  |  | - | *Penaeus monodon* | - | 6 |
| **60** | tensidol B / pestalamide A | - | Two cocoa (*Theobroma cacao* L.) bean (F and T series) hybrids | - | 2 |
|  |  | IBT 28144  (CBS 101705) | - | - | 20 |
|  |  | FGSC A1279 ΔgcnE mutant | - | - | 12 |
|  |  | - | *Penaeus monodon* | - | 6 |

**2. Alkaloids**

**2.1 Pyrrole analogs**

| **Compound NO.** | **Name** | **Strain** | **Source** | **Bioactivity** | **Ref.** |
| --- | --- | --- | --- | --- | --- |
| **61** | pyranonigrin B | - | Mediterranean sponge *Axinella damicornis* | - | 31 |
|  |  | - | *Penaeus monodon* | - | 6 |
| **62** | pyranonigrin C | - | Mediterranean sponge *Axinella damicornis* | - | 31 |
|  |  | - | *Penaeus monodon* | - | 6 |
| **63** | pyranonigrin A_b_ | - | Mediterranean sponge *Axinella damicornis* | Growth-inhibiting effect toward neonate larvae of insect | 31 |
|  |  | IBT 28144  (CBS 101705) | - | - | 20 |
|  |  | - | *Penaeus monodon* | - | 6 |
| **64** | pyranonigrin D | - | Mediterranean sponge *Axinella damicornis* | - | 31 |
| **65** | pyranonigrin A | NBRC5374 | - | Antioxidative activity(DPPH and superoxide) | 36, 37 |
| **66** | pyranonigrin S | NBRC5374 | - | Radical scavenging activity (DPPH) | 36 |
| **67** | pyranonigrin E(C_11_H_11_NO_4_) | NBRC5374 | - | Radical scavenging activity (DPPH) | 36 |
| **68** | pyranonigrin E(C_18_H_21_NO_4_) | ATCC 1015 | - | - | 38 |
| **69** | pyranonigrin F | ATCC 1015 | - | - | 38 |
| **70** | pyoluteorin | - | Soil | Cytotoxicity(MDA-MB-231cell line) | 21 |
| **71** | tensidol A | FKI-2342 | Soil(Nagasaki, Japan) | Potentiate miconazole activity; antimicrobial activity(*P. oryzae* and *C. albicans*) | 39 |
|  |  | ATCC 1015 | - | Antifungal activity(*C. strains, A. flavus* and *A. fumigatus*) | 40 |
| **72** | tensidol B_b_ | FKI-2342 | Soil(Nagasaki, Japan) | Potentiate miconazole activity; moderate antimicrobial activity(*P. oryzae* and *C. albicans*) | 39 |
|  |  | ATCC 1015 | - | Antifungal activity(*C. strains, A. flavus* and *A. fumigatus*) | 40 |

**2.2 Pyridones**

| **Compound NO.** | **Name** | **Strain** | **Source** | **Bioactivity** | **Ref.** |
| --- | --- | --- | --- | --- | --- |
| **73** | aspernigrin A | - | Mediterranean sponge *Axinella damicornis* | Cytotoxicity(leukemia and carcinoma cell lines) | 31 |
| **74** | aspernigrin B_b_ | - | Mediterranean sponge *Axinella damicornis* | Neuroprotective activity(glutamic acid), cytotoxicity(leukemia and carcinoma cell lines) | 31 |
| **75** | nygerone B | ATCC 1015 | - | Antifungal activity(*C. strains, A. flavus* and *A. fumigatus*) | 41, 42, 43 |
| **76** | aspernigrin A_b_ | SCSIO Jcsw6F30 | Marine alga *Sargassum* sp. | - | 1 |
| **77** | *p*-ﬂuoro nygerone B | ATCC 1015 | - | Antifungal activity(*C. strains, A. flavus* and *A. fumigatus*) | 40 |
| **78** | nygerone A | ATCC 1015 | - | Antifungal activity(*C. strains, A. flavus* and *A. fumigatus*) | 41, 42, 43 |
| **79** | pestalamide B | ATCC 11414 | - | - | 19 |
| **80** | aspernigrin B_a_ | SCSIO Jcsw6F30 | Marine alga *Sargassum* sp. | - | 1 |
| **81** | aspernigrin C | SCSIO Jcsw6F30 | Marine alga *Sargassum* sp. | Anti-HIV-1 activity | 1 |
| **82** | aspernigrin D | SCSIO Jcsw6F30 | Marine alga *Sargassum* sp. | - | 1 |

**2.3 Other alkaloids**

| **Compound NO.** | **Name** | **Strain** | **Source** | **Bioactivity** | **Ref.** |
| --- | --- | --- | --- | --- | --- |
| **83** | fumonisin B_2_ | FGSC A1279 | - | - | 35 |
|  |  | IBT 28144  (CBS 101705) | - | - | 20 |
|  |  | - | - | Carcinogenic activity(humans) | 44 |
| **84** | fumonisin B_1_ | FGSC A1279 | - | - | 35 |
| **85** | fumonisin B_4_ | IBT 28144  (CBS 101705) | - | - | 20 |
|  |  | - | - | Carcinogenic activity(humans) | 44 |
| **86** | azanigerone D | T1 | - | - | 45 |
| **87** | phenazine-1-carboxylic acid | - | Soil |  | 21 |
| **88** | nigragillin | - | Two cocoa (*Theobroma cacao* L.) bean (F and T series) hybrids |  | 2 |
|  |  | ATCC 11414 | - | - | 19 |
| **89** | nigerazine B | ATCC 11414 | *-* | - | 19 |
| **90** | aspernigerin | IFB-E003 | *Cyndon dactylon* | Cytotoxicity(nasopharynyeal epidermoid KB, cervical carcinoma Hela, and colorectal carcinoma SW1116 cell lines) | 46 |

**3. Amides**

| **Compound NO.** | **Name** | **Strain** | **Source** | **Bioactivity** | **Ref.** |
| --- | --- | --- | --- | --- | --- |
| **91** | furan ester derivative | BRF-074 | Marine sediments | Cytotoxicity(HCT-116 cell line) | 47 |
| **92** | nigerloxin | CFR-W-105 | - | Enzyme inhibitor(LOX-1, RLAR); free radical scavenging activity(DPPH) | 48 |
|  |  | MTCC-5166 | - | - | 49 |
| **93** | chrysogeside D | - | Soil |  | 21 |
| **94** | ergosterimide | EN-13 | Marine brown alga *Colpomenia sinuosa* | - | 50 |
| **95** | pseurotin D | BRF-074 | Marine sediments | - | 47 |
| **96** | pseurotin A | BRF-074 | Marine sediments | - | 47 |

**4. Cyclopeptides**

| **Compound NO.** | **Name** | **Strain** | **Source** | **Bioactivity** | **Ref.** |
| --- | --- | --- | --- | --- | --- |
| **97** | cyclo(*L*-Trp-*L*-Trp) | ­- | Liverwort *Heteroscyphus tener* (Steph.) Schiffn | - | 23 |
| **98** | cyclo (*L*-Trp-*L*-Ile) | EN-13 | Marine brown alga *Colpomenia sinuosa* | - | 26 |
| **99** | cyclo (*D*-Phe-*L*-Trp) /  cyclo (*L*-Trp-*L*-Phe) | - | Liverwort *Heteroscyphus tener* (Steph.) Schiffn | - | 23 |
|  |  | EN-13 | Marine brown alga *Colpomenia sinuosa* | - | 26 |
| **100** | cyclo (*L*-Trp-L-Tyr) | EN-13 | Marine brown alga *Colpomenia sinuosa* | - | 26 |
| **101** | cyclo (trans-4-hydroxy-*L*-Pro-*L*-Leu) | BRF-074 | Marine sediments | - | 47 |
| **102** | cyclo (trans-4-hydroxy-*L*-Pro-*L*-Phe) | BRF-074 | Marine sediments | - | 47 |
| **103** | cyclo (*L*-Pro-*L*-Leu) | BRF-074 | Marine sediments | - | 47 |
| **104** | cyclo (*L*-Pro-*L*-Phe) | BRF-074 | Marine sediments | - | 47 |
| **105** | cyclo (*L*-Pro-*L*-Tyr) | BRF-074 | Marine sediments | - | 47 |
| **106** | cyclo (*L*-Pro-*L*-Val) | BRF-074 | Marine sediments | - | 47 |
| **107** | malformin A / A_1_ / B_1_ / B_1a_ | - | Two cocoa (*Theobroma cacao* L.) bean (F and T series) hybrids | - | 2 |
|  |  | BRF-074 | Marine sediments | - | 47 |
|  |  | IBT 28144  (CBS 101705) | - | - | 20 |
|  |  | MA-132 | Mangrove plant *Avicennia marina* | Antibacterial activity(*S. aureus*) | 51 |
|  |  | 203 | Air | Mammalian toxicity(mice) | 52 |
|  |  | 56-39 | - | - | 53 |
|  |  | 56-30 | - | - | 54 |
| **108** | malformin A_2_ | 56-39 | - | - | 53 |
| **109** | malformin A_4_ | 56-39 | - | - | 53 |
| **110** | malformin B_2_ | 56-30 | - | - | 54 |
| **111** | malformin B_3_ | - | - | - | 55 |
| **112** | malformin B_4_ | - | - | - | 55 |
| **113** | malformin B_5_ | - | - | - | 55 |
| **114** | malformin C / B_1b_ / A_3_ | SCSIO Jcsw6F30 | Marine alga *Sargassum* sp. | Anti-HIV-1 activity | 1 |
|  |  | - | Two cocoa (*Theobroma cacao* L.) bean (F and T series) hybrids | - | 2 |
|  |  | 15F41-1-3 | Unidentified marine sponge | Cytotoxicity(DU145 cells) | 7 |
|  |  | BRF-074 | Marine sediments | - | 47 |
|  |  | 94-1212 | *Hyrtios proteus* sponge |  | 27, 28 |
|  |  | IBT 28144  (CBS 101705) |  |  | 20 |
|  |  | MA-132 | Mangrove plant *Avicennia marina* | Antibacterial activity(*S. aureus*) | 51 |
|  |  | 56-39 |  |  | 53 |
|  |  | AN-1 |  |  | 56 |
| **115** | asperazine | - | Soil | - | 21 |
|  |  | - | Liverwort *Heteroscyphus tener* (Steph.) Schiffn | Cytotoxicity(A2780 cell lines) | 23 |
|  |  | 94-1212 | *Hyrtios proteus* sponge | Cytotoxicity(human leukemia murine colon 38 and human colon H116 or CX1 cell lines.) | 27, 28 |
| **116** | asperazine A | ­- | Liverwort *Heteroscyphus tener* (Steph.) Schiffn | Cytotoxicity(A2780 cell lines) | 23 |
| **117** | diketopiperazine dimer | - | Marine derived | - | 8 |

**5. Polyketides**

| **Compound NO.** | **Name** | **Strain** | **Source** | **Bioactivity** | | **Ref.** | |
| --- | --- | --- | --- | --- | --- | --- | --- |
| **118** | citric acid | ATCC 1015 | - | - | | 57 | |
| **119** | itaconic acid | CAD | - | - | | 58 | |
| **120** | hexylitaconic acid | 94-1212 | *Hyrtios proteus* sponge | - | | 27, 28 | |
| **121** | penitricin D | AM410 | Soil(near Perth, Western Australia) | Enzyme inhibitor(CD45 tyrosine phosphatase) | | 59 | |
| **122** | asperic acid | 94-1212 | *Hyrtios proteus* sponge | - | | 27 | |
| **123** | carlosic acid | ATCC 1015 | - | - | | 60 | |
| **124** | carlosic acid methyl ester | ATCC 1015 | - | - | | 60 | |
| **125** | agglomerin F | ATCC 1015 | - | - | | 60 | |
| **126** | dihydrocarolic acid | AM410 | Soil(near Perth, Western Australia) | Enzyme inhibitor(CD45 tyrosine phosphatase) | | 59 | |
| **127** | chlovalicin | BRF-074 | Marine sediments | - | | 47 | |
| **128** | 2-phenylethanol | DSM821 | - | - | | 61 | |
| **129** | *p*-hydroxyphenylacetic acid | AKRN | Roots of *Entandrophragma congoënse* | - | | 22 | |
| **130** | gallic acid | FCPB-SF-0002 | - | - | | 62 | |
| **131** | FK17-p2a | T1 | - | - | | 45 | |
| **132** | yanuthone I | KB1001 | - | - | 63 | | |
| **133** | yanuthone J | KB1001 | - | - | 63 | | |
| **134** | yanuthone B | F97S11 | Orange ascidian *Aplidium* sp. | Antimicrobial activity(methicillin sensitive *S. aureus*) | 64 | | |
| **135** | yanuthone D | KB1001 | - | Antifungal activity(*C. albicans*) | 63 | | |
|  |  | F97S11 | Orange ascidian *Aplidium* sp. | Antimicrobial activity(methicillin sensitive *S. aureus*) | 64 | | |
| **136** | 7-deacetoxyyanuthone A | KB1001 | - | - | 63 | | |
| **137** | yanuthone A | F97S11 | Orange ascidian *Aplidium* sp. | Antimicrobial activity(methicillin sensitive S. aureus) | 64 | | |
| **138** | yanuthone C | F97S11 | Orange ascidian *Aplidium* sp. | Antimicrobial activity(methicillin sensitive *S. aureus*) | 64 | | |
| **139** | yanuthone E | KB1001 | - | Antifungal activity(*C. albicans*) | 63 | | |
|  |  | F97S11 | Orange ascidian *Aplidium* sp. | ,Antimicrobial activity(methicillin sensitive *S. aureus*) | 64 | | |
| **140** | yanuthone F | KB1001 | - | Antimicrobial activity(methicillin sensitive *S. aureus*) | 64 | | |
| **141** | yanuthone G | KB1001 | - | Antimicrobial activity(*C. albicans*) | 63 | | |
| **142** | yanuthone H | KB1001 |  | Antimicrobial activity(*C. albicans*) | 63 | | |
| **143** | 1-hydroxyyanuthone A | F97S11 | Orange ascidian *Aplidium* sp. | Antimicrobial activity(methicillin sensitive *S. aureus*) | 64 | | |
| **144** | 1-hydroxyyanuthone C | F97S11 | Orange ascidian *Aplidium* sp. | Antimicrobial activity(methicillin sensitive *S. aureus*) | 64 | | |
| **145** | 22-deacetylyanuthone A | KB1001 | - | Antifungal activity | 63 | | |
|  |  | F97S11 | Orange ascidian *Aplidium* sp. | Antimicrobial activity | 64 | | |
| **146** | yanuthone X_1_ | KB1001 | - | - | 63 | | |
| **147** | asperyellone / asperenone | AN01, NRRL-3 | - | - | | 65 | |
|  |  | CFTRI 1105 | - | - | | 66 | |
| **148** | flaviolin | - | - | - | | 67 |  |
| **149** | azanigerone A | T1 | - | - | | 45 | |
| **150** | funalenone | FGSC A1279 ΔgcnE mutant | - | - | | 12 | |
|  |  | ATCC 11414 | - | - | | 19 | |
| **151** | azanigerone B | T1 | - | - | | 45 | |
| **152** | azanigerone C | T1 | - | - | | 45 | |
| **153** | azanigerone E | T1 | - | - | | 45 | |
| **154** | azanigerone F | T1 | - | - | | 45 | |
| **155** | aspulvinone E | - | Two cocoa (*Theobroma cacao* L.) bean (F and T series) hybrids | - | | 2 | |
| **156** | cycloleucomelone | - | Mediterranean sponge *Axinella damicornis* | - | | 31 |  |
| **157** | BMS-192548 | WB2346 | Soil (Honolulu, Hawaii) | Cytotoxicity(M-109 cell line), Neuropeptide Y and neurokinin-1 receptors antagonist | | 68, 69 | |
| **158** | TAN-1612 | ATCC 1015 | - | - | | 70 | |

**6. Sterols**

| **Compound NO.** | **Name** | **Strain** | **Source** | **Bioactivity** | **Ref.** |
| --- | --- | --- | --- | --- | --- |
| **159** | 14-dehydroergosterol | - | - | - | 71 |
| **160** | 14-dehydroergosteryl benzoate | - | - | - | 71 |
| **161** | nigerasterol A | MA-132 | Mangrove plant *Avicennia marina* | Antiproliferative Activity(HL60 and A549 cell lines) | 51 |
| **162** | nigerasterol B | MA-132 | Mangrove plant *Avicennia marina* | Antiproliferative Activity(HL60 and A549 cell lines) | 51 |
| **163** | ergosterol peroxide | - | Soil | - | 21 |
|  |  | EN-13 | Marine brown alga *Colpomenia sinuosa* | - | 50 |
| **164** | (22*E*,24*R*)-ergosta-5,7,22-trien-3*β*-ol | EN-13 | Marine brown alga *Colpomenia sinuosa* | - | 50 |
| **165** | (22*E*,24*R*)-ergosta-4,6,8(14),22-tetraen-3-one | EN-13 | Marine brown alga *Colpomenia sinuosa* | - | 50 |
| **166** | (22*E*,24*R*)-ergosta-7,22-dien-3*β*,5*α*,6*β*-triol | EN-13 | Marine brown alga *Colpomenia sinuosa* | - | 50 |

**REFERENCE**

1. Zhou, X.; Fang, W.; Tan, S.; Lin, X.; Xun, T.; Yang, B.; Liu, S.; Liu, Y. Aspernigrins with anti-HIV-1 activities from the marine-derived fungus *Aspergillus niger* SCSIO Jcsw6F30, *Bioorg. Med. Chem. Lett.*, 2015, 26(2), 361-365. doi:10.1016/j.bmcl.2015.12.005-

2. Akinfala T.O.; Houbraken J.; Sulyok M. et al., Moulds and their secondary metabolites associated with the fermentation and storage of two cocoa bean hybrids in Nigeria, *Int. J. of Food Microbiol.* , 2019, doi:10.1016/j.ijfoodmicro.2019.108490

3. Sakurai, M.; Kohno, J.; Yamamoto, K.; Okuda, T.; Nishio, M.; Kawano, K.; Ohnuki, T. TMC-256A1 and C1, New inhibitors of IL-4 signal transduction produced by *Aspergillus niger* var niger TC 1629. *J. Antibiot*., 2002, 55(8), 685-692. doi:10.7164/antibiotics.55.685

4. Leutou, A. S.; Yun, K.; Son, B. W. Induced production of 6,9-dibromoflavasperone, a new radical scavenging naphthopyranone in the marine-mudflat-derived fungus *Aspergillus niger*. *Arch. Pharm. Res*., 2016, 39(6), 806-810. doi:10.1007/s12272-016-0764-2

5. Bouras, N.; Mathieu, F.; Coppel, Y.; Lebrihi, A. Aurasperone F - a new member of the naphtho-gamma-pyrone class isolated from a cultured microfungus, *Aspergillus niger* C-433. *Nat. Prod. Res.*, 2005, 19(7), 653-659. doi:10.1080/1478641041233128695

6. Fernand, M. G.; Roullier, C.; Guitton, Y.; Lalande, J.; Lacoste, S.; Dupont, J.; Ranaivoson, E. Fungi isolated from Madagascar shrimps - investigation of the *Aspergillus niger* metabolism by combined LC-MS and NMR metabolomics studies. *Aquaculture*, 2017, 479, 750-758. doi:10.1016/j.aquaculture.2017.07.015

7. Jomori, T.; Hara, Y.; Sasaoka, M. *et al.* Mycobacterium smegmatis alters the production of secondary metabolites by marine-derived *Aspergillus niger. J. Nat. Med-TOKYO* 2019, doi:10.1007/s11418-019-01345-0

8. Ovenden, S.P.B.; Sberna, G.; Tait, R.M.; Wildman, H.G.; Patel, R.; Li, B.; Meurer G.B.M. A Diketopiperazine dimer from a marine-derived isolate of *Aspergillus niger*. *J. Nat. Prod.,* 2004, 67(12), 2093-2095. doi:10.1021/np0497494

9. Zhang Y.; Li X.M.; Wang B.G. Nigerasperones A~C, New monomeric and dimeric naphthogγ-pyrones from a marine alga-derived endophytic fungus *Aspergillus niger* EN-13. *J. Antibiot.*, 2007, 60(3): 204-210

10. Song, Y.C.; Li, H.; Ye, Y.H.; Shan, C.Y.; Yang, Y.M.; Tan, R.X. Endophytic naphtha pyrone metabolites are co-inhibitors of xanthine oxidase, SW1116 cell and some microbial growths. *FEMS Microbiol. Lett.*, 2004, 241(1), 67-72. doi:10.1016/j.femsle.2004.10.005

11. Li, D.H.; Han, T.; Guan, L.P.; Bai, J.; Zhao, N.; Li, Z.L.; Hua, H.-M. New naphthopyrones from marine-derived fungus *Aspergillus niger* 2HL-M-8 and their in vitroantiproliferative activity. *Nat. Prod. Res.*, 2015, 30(10), 1116-1122. doi:10.1080/14786419.2015.1043553

12. Wang, B.; Li, X.; Yu, D.; Chen, X.; Tabudravu, J.; Deng, H.; Li, P. Deletion of the epigenetic regulator *Gcn*E in *Aspergillus niger* FGSC A1279 activates the production of multiple polyketide metabolites. *Microbiol. Res.,* 2018, doi:10.1016/j.micres.2018.10.004

13. Tanaka, H.; Wang, P.L.; Yamada, L.O.; Tamura, T. Yellow pigments of *Aspergillus niger* and *A. sp.* awamori. *Agricultural and Biological Chemistry*, 1966, 30(2), 107-113. doi:10.1080/00021369.1966.10858561

14. Tanaka, H.; Pie, L.W.; Namiki, M. Structure of Aurasperone C. *Agricultural and Biological Chemistry*, 1972, 36(13), 2511-2517. doi:10.1080/00021369.1972.10860562

15. Wang, P.L.; Tanaka, H. Yellow Pigments of *Aspergillus niger* and *Aspergillus awamori*. *Agric. Biol. Chem.* 1966, 30(7), 683-687. doi:10.1080/00021369.1966.10858657

16. Fang, W.; Lin, X.; Wang, J.; Liu, Y.; Tao, H.; Zhou, X. Asperpyrone-Type Bis-Naphtho-γ-Pyrones with COX-2–Inhibitory activities from marine-derived fungus *Aspergillus niger*. *Molecules,* 2016, 21(7), 941. doi:10.3390/molecules21070941

17. Akiyama, K.; Teraguchi, S.; Hamasaki, Y.; Mori, M.; Tatsumi, K.; Ohnishi, K.; Hayashi, H. New dimeric naphthopyrones from *Aspergillus niger*. *J. Nat. Prod.*, 2003, 66(1), 136-139. doi:10.1021/np020174p

18. Padhi, S.; Masi, M.; Panda, S. K.; Luyten, W.; Cimmino, A.; Tayung, K.; Evidente, A. Antimicrobial secondary metabolites of an endolichenic *Aspergillus niger* isolated from lichen thallus of *Parmotrema ravum*. *Nat. Prod. Res.*, 2019, 1-8. doi:10.1080/14786419.2018.1544982

19. Chiang Y. M.; Meyer K.M.; Praseuth M. *et al*. Characterization of a polyketide synthase in *Aspergillus niger* whose product is a precursor for both dihydroxynaphthalene (DHN) melanin and naphtho-γ-pyrone. *Fungal Genet. Biol.* 2011, 48(4), 430-437. doi:10.1016/j.fgb.2010.12.001

20. Sørensen L.M.; Lametsch R.; Andersen M.R. et al. Proteome analysis of *Aspergillus niger*: Lactate added in starch-containing medium can increase production of the mycotoxin fumonisin B2 by modifying acetyl-CoA metabolism. *BMC Microbiol.* 2009, *9(1), 255.* doi:10.1186/1471-2180-9-255

21. Ding T.; Yang L.J.; Zhang W. D. *et al.* Pyoluteorin induces cell cycle arrest and apoptosis in human triple-negative breast cancer cells MDA-MB-231. *J. Pharm. Pharmacol.* 2020, 10.1111/jphp.13262

22. Happi, G.M.; Kouam, S.F.; Talontsi, F.M.; Nkenfou, C.N.; Longo, F.; Zühlke, S.; Spiteller, M. A new dimeric naphtho-γ-pyrone from an endophytic fungus *Aspergillus niger* AKRN associated with the roots of Entandrophragma congoënse collected in Cameroon. *Z. Naturforsch, B: J, Che. Sci.*, 2015, 70(9), 625-630. doi:10.1515/znb-2015-0036

23. Li, X.B.; Li, Y.L.; Zhou, J.C.; Yuan, H.Q.; Wang, X.N.; Lou, H.X. A new diketopiperazine heterodimer from an endophytic fungus *Aspergillus niger*. *J. Asian Nat. Prod. Res.*, 2014, 17(2), 182-187. doi:10.1080/10286020.2014.959939

24. Liu, D.; Li, X.M.; Meng, L.; Li, C.S.; Gao, S.S.; Shang, Z.; Wang, B.G. Nigerapyrones A-H, *α*-pyrone derivatives from the marine mangrove-derived endophytic fungus *Aspergillus niger* MA-132. *J. Nat. Prod.*, 2011, 74(8), 1787-1791. doi:10.1021/np200381u

25. Mouafo T.F.; Kongue T. M. D.; Dittrich, B.; Douanla, M.C.; Laatsch, H. Structures and absolute configuration of three *α*-pyrones from an endophytic fungus *Aspergillus niger*. *Tetrahedron*, 2013, 69(34), 7147-7151. doi:10.1016/j.tet.2013.05.098

26. Zhang, Y.; Li, X.M.; Feng, Y.; Wang, B.G. Phenethyl-α-pyrone derivatives and cyclodipeptides from a marine algous endophytic fungus *Aspergillus niger* EN-13. *Nat. Prod. Res.*, 2010, 24(11), 1036-1043. doi:10.1080/14786410902940875

27. Varoglu, M.; Crews, P. Biosynthetically diverse compounds from a saltwater culture of sponge-derived *Aspergillus niger*. *J. Nat. Prod.*, 2000, 63(1), 41-43. doi:10.1021/np9902892

28. Varoglu, M.; Corbett, T. H.; Valeriote, F. A.; Crews, P. Asperazine, a selective cytotoxic alkaloid from a sponge-derived culture of *Aspergillus niger*. *J. Org. Chem.*, 1997, 62(21), 7078-7079. doi:10.1021/jo970568z

29. Takano, D. Absolute conﬁguration of nafuredin, a new speciﬁc NADH-fumarate reductase inhibitor. *Tetrahedron Lett.*, 2001, 42: 3017-3020.

30. Ui, H.; Shiomi, K.; Yamaguchi, Y.; Masuma, R.; Nagamitsu, T.; Takanol, D.; Sunazuka, T.; Namikoshi, M.; Omura, S. Nafuredin, a novel inhibitor of NADH-fumarate Reductase, produced by *Aspergillus niger* FT-0554. *J. Antibiot.*, 2001, 54 (3), 234-238.

31. Hiort, J.; Maksimenka, K.; Reichert, M.; Perović-Ottstadt, S.; Lin, W. H.; Wray, V.; Bringmann, G. new natural products from the sponge-derived fungus *Aspergillus niger*. *J. Nat. Prod.*, 2004, 67(9), 1532-1543. doi:10.1021/np030551d

32. Gil G., C.; Fisch, K.M.; Heinekamp, T.; Günther, S.; Hüttel, W.; Piel, J.; Müller, M. Regio- and stereoselective oxidative phenol coupling in *Aspergillus niger*. *Angewandte Chemie-International Edition*, 2012, 51(39), 9788-9791. doi:10.1002/anie.201203603

33. Cutler, H.G.; Crumley, F.G.; Cox, R.H.; Hernandez, O.; Cole, R.J.; Dorner, J.W. Orlandin: a nontoxic fungal metabolite with plant growth inhibiting properties. *J. Agr. Food Chem.*, 1979, 27(3), 592-595. doi:10.1021/jf60223a043

34. Copetti M.V.; Pereira J.L.; Iamanaka B.T. *et al.* Ochratoxigenic fungi and ochratoxin A in cocoa during farm processing. *Int. J. Food Microbiol.* 2010, 143(1-2), 67-70*.* doi:10.1016/j.ijfoodmicro.2010.07.031

35. Li X.; Pan L.; Wang B. *et al.* The histone deacetylases *HosA* and *HdaA* affect the phenotype and transcriptomic and metabolic profiles of *Aspergillus niger. Toxins,* 2019, 11(9), 520. doi:10.3390/toxins11090520

36. Riko, R.; Nakamura, H.; Shindo, K. Studies on pyranonigrins–isolation of pyranonigrin E and biosynthetic studies on pyranonigrin A. *J. Antibiot.*, 2013, 67(2), 179-181. doi:10.1038/ja.2013.91

37. Miyake Y.; Ito C.; Itoigawa M. *et al.* Isolation of the antioxidant pyranonigrin-A from rice mold starters used in the manufacturing process of fermented foods*. Biosci. Biotech. Bioch.,* 2007, 71(10), 2515-2521. doi:10.1271/bbb.70310

38. Yamamoto T.; Tsunematsu Y.; Noguchi, H. *et al.* Elucidation of pyranonigrin biosynthetic pathway reveals a mode of tetramic acid, fused γ-pyrone, and exo-methylene formation. *Org. Lett.,* 2015, 17(20), 4992-4995. doi:10.1021/acs.orglett.5b02435

39. Fukuda T.; Hasegawa Y.; Hagimori K. *et al.* Tensidols, new potentiators of antifungal miconazole activity, produced by *Aspergillus niger* FKI-2342*. J. Antibiot*., 2006, 59(8), 480-485. doi:10.1038/ja.2006.67

40. Henrikson, J. C.; Ellis, T. K.; King, J. B.; Cichewicz, R. H. Reappraising the structures and distribution of metabolites from black Aspergilli containing uncommon 2-Benzyl-4H-pyran-4-one and 2-Benzylpyridin-4(1H)-one systems. *J. Nat. Prod.*, 2011, 74(9), 1959–1964. doi:10.1021/np200454z

41. Henrikson J.C.; Hoover A.R.; Joyner P.M. *et al.* A chemical epigenetics approach for engineering the in situbiosynthesis of a cryptic natural product from *Aspergillus niger. Org. Biomol. Chem.,* 2009, 7(3), 435-438. doi:10.1039/b819208a

42. Fisch, K. M.; Gillaspy, A. F.; Gipson, M.; Henrikson, J. C.; Hoover, A. R.; Jackson, L.; Cichewicz, R. H. Chemical induction of silent biosynthetic pathway transcription in Aspergillus niger. *J. Ind. Microbiol. Biot.*, 2009, 36(9), 1199-1213. doi:10.1007/s10295-009-0601-4

43. Williams R.B.; Henrikson J.C.; Hoover A.R. *et al.* Epigenetic remodeling of the fungal secondary metabolome. *Org. Biomol. Chem.,* 2008, 6(11), 1895. doi:10.1039/b804701d

44. Nielsen, K.F.; Mogensen, J.M.; Johansen, M.; Larsen, T.O.; Frisvad, J.C. Review of secondary metabolites and mycotoxins from the *Aspergillus niger* group. *Anal. Bioanal. Chem.*, 2009, 395(5), 1225-1242. doi:10.1007/s00216-009-3081-5

45. Zabala A.O.; Xu W.; Chooi Y.H. *et al.* Characterization of a silent azaphilone gene cluster from *Aspergillus niger* ATCC 1015 reveals a hydroxylation-mediated pyran-ring formation. *Chem. Biol.,* 2012, 19(8), 1049-1059. doi:10.1016/j.chembiol.2012.07.004

46. Shen, L.; Ye, Y.H.; Wang, X.T.; Zhu, H.L.; Xu, C.; Song, Y.C.; Tan, R.X. Structure and total synthesis of Aspernigerin: A novel cytotoxic endophyte metabolite. *Chemistry - A European Journal*, 2006, 12(16), 4393-4396. doi:10.1002/chem.200501423

47. Uchoa, P.K.S.; Pimenta, A.T.A.; Braz, F.R.; de Oliveira, M.; da C.F.; Saraiva, N.N.; Rodrigues, B.S. F.; Lima, M.A.S. New cytotoxic furan from the marine sediment-derived fungi Aspergillus niger. *Nat. Prod. Res.*, 2017, 31(22), 2599-2603. doi:10.1080/14786419.2017.1283499

48. Rao, K.C.S.; Divakar, S.; Babu, K.N.; Rao, A.G.A.; Karanth, N.G.; Sattur, A.P. Nigerloxin, a novel inhibitor of aldose reductase and lipoxygenase with free radical scavenging activity from *Aspergillus niger* CFR-W-105. *ChemInform*, 2002, 34(10). doi:10.1002/chin.200310218

49. Chakradhar, D.; Javeed, S.; Sattur, A. P. Studies on the production of nigerloxin using agro-industrial residues by solid-state fermentation. *J. Ind. Microbiol. Biot.*, 2009, 36(9), 1179-1187. doi:10.1007/s10295-009-0599-7

50. Zhang, Y.; Li, X.M.; Proksch, P.; Wang, B.G. Ergosterimide, a new natural diels–alder adduct of a steroid and maleimide in the fungus *Aspergillus niger*. *Steroids*, 2007, 72(9-10), 723-727. doi:10.1016/j.steroids.2007.05.009

51. Liu, D.; Li, X.-M.; Li, C.S.; Wang, B.G. Nigerasterols A and B, Antiproliferative sterols from the mangrove-derived endophytic fungus *Aspergillus niger* MA-132. *Helvetica. Chimica. Acta.*, 2013, 96(6), 1055–1061. doi:10.1002/hlca.201200332.

52. Yoshizawa T.; Tsuchiya Y.; Morooka N. *et al*. Malformin A_1_ as a mammalian toxicant from *Aspergillus niger*, *Agr. BioI. Chem.*, 1975, 39 (6), 1325-1326.

53. John, W.W. and Curtis R.W. Stimulation of plant growth by malformin A. *Experientia*, 1974, 30, 1392-1393.

54. Takahashi N. and Curtis R.W. Isolation and characterization of malformin, *Plant Physiol*., 1961, 30-36

55. Kim K.W.; Sugawara F.; Yoshida S. *et al*. Structure of malformin B, a phytotoxic metabolite produced by *Aspergillus niger. Biosci. Biotech. Bioch.,* 1993, 57(5), 787-791. doi:10.1271/bbb.57.787

56.Takeuchi, S.; Senn, M.; Curtis, R. W.; McLafferty, F. W. Chemical studies on Malformin-V. *Phytochemistry*, 1967, 6(2), 287-292. doi:10.1016/s0031-9422(00)82774-0

57. Andersen M.R.; Salazar M.P.; Schaap P.J. et al. Comparative genomics of citric-acid-producing *Aspergillus niger* ATCC 1015 versus enzyme-producing CBS 513.88. *Genome Res*., 2011, 21(6), 885-897. doi:10.1101/gr.112169.110

58. Li, A.; Pfelzer, N.; Zuijderwijk, R.; Punt, P. Enhanced itaconic acid production in *Aspergillus niger* using genetic modification and medium optimization. *BMC Biotechnol.*, 2012, 12(1), 57. doi:10.1186/1472-6750-12-57

59. Alvi, K.A.; NAIR, B.G.; RABENSTEIN, J.; DAVIS, G.; BAKER, D.D. CD45 Tyrosine phosphatase inhibitory components from *Aspergillus niger*. *J. Antibiot.*, 2000, 53(2), 110-113. doi:10.7164/antibiotics.53.110

60. Yang, X.L.; Awakawa, T.; Wakimoto, T.; Abe, I. Three acyltetronic acid derivatives: noncanonical cryptic polyketides from *Aspergillus niger* identified by genome mining. *Chem. Bio. Chem.* 2014, 15(11), 1578-1583. doi:10.1002/cbic.201402172

61. Etschmann, M.M.W.; Huth, I.; Walisko, R.; Schuster, J.; Krull, R.; Holtmann, D.; Schrader, J. Improving 2-phenylethanol and 6-pentyl-α-pyrone production with fungi by microparticle-enhanced cultivation (MPEC). *Yeast*, 2014. doi:10.1002/yea.3022

62. Saeed S.; Aslam S.; Mehmood T. *et al.* Production of gallic acid under solid-state fermentation by utilizing waste from food processing industries. *waste biomass valorization.* 2020, doi:10.1007/s12649-020-00980-z

63. Holm D.K.; Petersen L.M.; Klitgaard A. *et al*. Molecular and chemical characterization of the biosynthesis of the 6-MSA-derived meroterpenoid yanuthone D in *Aspergillus niger*. *Chem. Biol.*, 2014, 21(4), 519-529. doi:10.1016/j.chembiol.2014.01.013

64. Bugni T.S.; Abbanat D.; Bernan V.S. *et al*. Yanuthones:  novel metabolites from a marine isolate of *Aspergillus niger. J. Org. Chem*. 2000, 65(21), 7195-7200. doi:10.1021/jo0006831

65. Jefferson, W.E. The isolation and characterization of asperenone, a new phenylpolyene from *Aspergillus niger*. *Biochemistry*, 1967, 6(11), 3479-3484. doi:10.1021/bi00863a019

66. Chidananda, C.; Kumar, C.M.; Sattur, A.P. Strain improvement of *Aspergillus niger* for the enhanced production of asperenone. *Indian J. Microbiol.*, 2008, 48(2), 274–278. doi:10.1007/s12088-008-0026-1

67. McGovern E.P.; Bentley R. Biosynthesis of flaviolin and 5,8-Dihydroxy-2,7-dimethoxy- 1,4-naphthoquinone, *Biochemistry-US*, 1975, 14(14), 3138-3143.

68. Kodukula K.; Arcuri M.; Cutrone J.Q. *et al*. BMS-192548, a tetracyclic binding inhibitor of neuropeptide Y receptors, from *Aspergillus niger* WB2346. I. Taxonomy, fermentation, isolation and biological activity. *J. Antibiot.*, 1995, 48(10), 1055-1059. doi:10.7164/antibiotics.48.1055

69. Shu Y.Z.; Cutrone J.Q.; Klohr S.E. *et al*. BMS-192548, a Tetracyclic Binding Inhibitor of Neuropeptide Y Receptors, from *Aspergillus niger* WB2346. II. Physico-chemical properties and structural characterization. *J. Antibiot.*, 1995, 48(10), 1060-1065. doi:10.7164/antibiotics.48.1060

70. Li Y.; Chooi Y.H.; Sheng Y.W. *et al.* Comparative characterization of fungal anthracenone and naphthacenedione biosynthetic pathways reveals an *α*-Hydroxylation-dependent claisen-like cyclization catalyzed by a dimanganese thioesterase. *J. Am. Chem. Soc*., 2011, 133(39), 15773-15785. doi:10.1021/ja206906d

71. Barton D.H.R.; Bruun T. A new sterol from a strain of *Aspergillus niger* . *J. Chem. Soc. Pakstan.* (Resumed), 1951, 2728. doi:10.1039/jr9510002728
